# Mutually exclusive acetylation and ubiquitylation of the splicing factor SRSF5 control tumor growth

**DOI:** 10.1038/s41467-018-04815-3

**Published:** 2018-06-25

**Authors:** Yuhan Chen, Qingyang Huang, Wen Liu, Qiong Zhu, Chun-Ping Cui, Liang Xu, Xing Guo, Ping Wang, Jingwen Liu, Guanglong Dong, Wenyi Wei, Cui Hua Liu, Zhichun Feng, Fuchu He, Lingqiang Zhang

**Affiliations:** 1 State Key Laboratory of Proteomics, Beijing Proteome Research Center, National Center of Protein Sciences (Beijing), Beijing Institute of Lifeomics, Beijing, 100850 China; 2 Department of Genomics and Proteomics, Beijing Institute of Radiation Medicine, Beijing, 100850 China; 3 Affiliated BaYi Children’s Hospital, PLA Army General Hospital, National Engineering Laboratory for Birth Defects Prevention and Control of Key Technology, Beijing Key Laboratory of Pediatric Organ Failure, Beijing, 100700 China; 4 Department of Biochemistry and Molecular Biology, Anhui Medical University, Hefei, 230032 Anhui China; 5 Department of Neurobiology, Key Laboratory of Human Functional Genomics of Jiangsu Province, Nanjing Medical University, Nanjing, 211166 Jiangsu China; 6 Department of Central Laboratory, Shanghai Tenth People’s Hospital, School of Life Science and Technology, Tongji University, Shanghai, 200072 China; 7 Department of General Surgery, Chinese People’s Liberation Army General Hospital, Beijing, 100853 China; 8 Department of Pathology, Beth Israel Deaconess Medical Center, Harvard Medical School, Boston, MA 02115 USA; 9 CAS Key Laboratory of Pathogenic Microbiology and Immunology, Institute of Microbiology, Chinese Academy of Sciences, Beijing, 100101 China; 10 School of Life Science, Jiangsu Normal University, Xuzhou, 221116 Jiangsu China

## Abstract

Most tumor cells take up more glucose than normal cells. Splicing dysregulation is one of the molecular hallmarks of cancer. However, the role of splicing factor in glucose metabolism and tumor development remains poorly defined. Here, we show that upon glucose intake, the splicing factor SRSF5 is specifically induced through Tip60-mediated acetylation on K125, which antagonizes Smurf1-mediated ubiquitylation. SRSF5 promotes the alternative splicing of *CCAR1* to produce CCAR1S proteins, which promote tumor growth by enhancing glucose consumption and acetyl-CoA production. Conversely, upon glucose starvation, SRSF5 is deacetylated by HDAC1, and ubiquitylated by Smurf1 on the same lysine, resulting in proteasomal degradation of SRSF5. The CCAR1L proteins accumulate to promote apoptosis. Importantly, SRSF5 is hyperacetylated and upregulated in human lung cancers, which correlates with increased *CCAR1S* expression and tumor progression. Thus, SRSF5 responds to high glucose to promote cancer development, and SRSF5–CCAR1 axis may be valuable targets for cancer therapeutics.

## Abstract (web summary)

Changes in glucose metabolism can lead to tumor development, but the involvement of splicing factors is unclear. Here, the authors screened for SR proteins and identified SRSF5 stability is enhanced in response to glucose elevation to promote alternative splicing of CCAR1 which facilitates tumor growth.

## Introduction

Emerging as one of the most prevalent mechanisms of gene regulation, alternative splicing (AS) plays a vital role in the intricate regulation of protein function and splicing dysregulation is closely associated with human cancers^1^. AS is mainly regulated by multiple *cis-elements* that recruit various splicing factors to the adjacent splicing site by distinct mechanisms^2^. Notably, the splicing factors can be divided into two categories, the serine/arginine (SR) proteins that promote splicing in a context-dependent manner and heterogeneous nuclear ribonucleoproteins (hnRNPs) that can both positively and negatively regulate splicing^3^. The SR proteins are composed of classical SR-splicing factors (SRSFs) and RNA binding SR-like splicing factors^4^. So far, all reported classical *SRSF* knockout mice displayed an early embryonic lethal phenotype^5–10^, thus supporting the fundamental roles of SR proteins in vivo and further suggesting that fine-tuning of abundance and activity of SRSFs determine splicing outcome in different cellular and organizational conditions.

Recent discoveries have demonstrated that dysregulation of SRSFs contributes to the progression of multiple types of human tumors^11^. For example, the proto-oncogene SRSF1 controls a myriad of genes in the key hubs of cancer signaling pathways, and the gain-of-function mutations of SRSF2 contribute to the development of myeloproliferative neoplasms^12,13^. Moreover, SRSF9 has been identified as an oncogenic transformer of colorectal cancers by promoting the accumulation of β-catenin^14^, and SRSF10 was shown to promote colorectal cancer progression by enhancing the splicing of anti-apoptosis isoform BCLAF1^15^. Since altered splicing is likely to pose a potential risk of cancers, specifically targeting SRSFs will provide novel insights into cancer therapies.

Dysregulation of cellular metabolism is a hallmark of cancer^16^, among which, the elevated glycolysis pathway plays guiding roles in facilitating tumor growth. Because glucose is the most important source for nutrient synthesis and can serve as building block for cell growth, most tumor cells take up more glucose than normal cells and the cellular responses to high glucose should contribute to the tumor development. Classical SR proteins have been currently reported to regulate metabolic homeostasis and energy-dependent development^17,18^. However, the role of splicing factors in glucose metabolism and tumor development still remains poorly defined.

Here, through a screen of SRSF family, we identified SRSF5 as a glucose-inducible protein that promotes tumor cell growth via AS of CCAR1, a master of cell cycle arrest and apoptosis. Interestingly, Tip60-mediated acetylation, HDAC1-mediated deacetylation and Smurf1-mediated ubiquitylation of SRSF5 on the common lysine residue orchestrate with each other to determine the cell fate in response to abundant or insufficient glucose. We also found that abnormal hyperacetylation of SRSF5 promotes the development of human lung cancer.

## Results

### SRSF5 is stabilized at high glucose to promote tumorigenesis

**Fig. 1:**
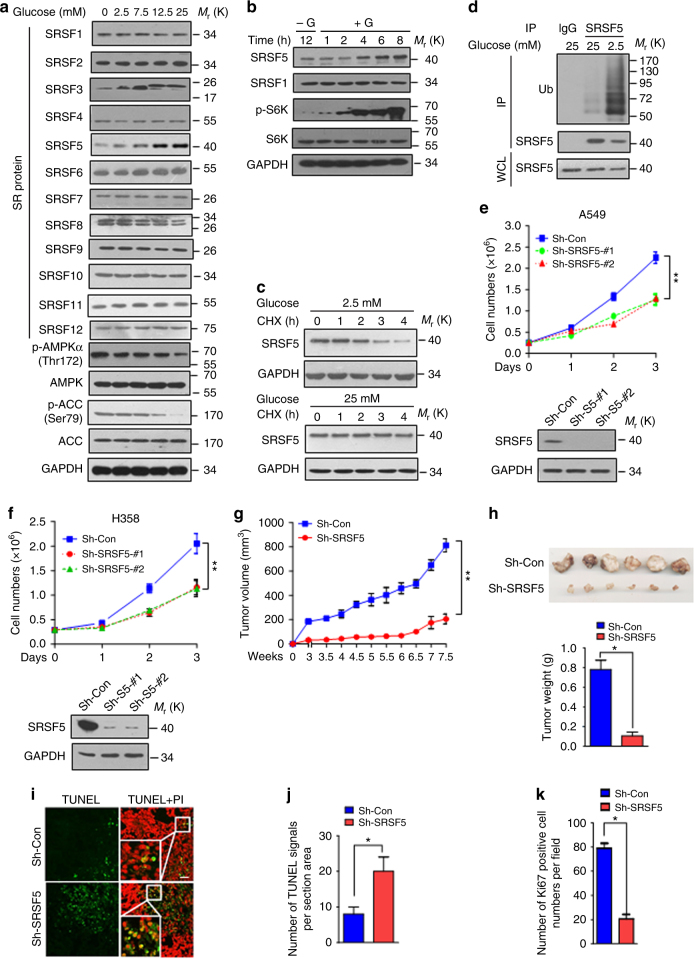
SRSF5 is stabilized at high glucose to promote tumorigenesis. **a** A549 cells were cultured in medium containing glucose with indicated concentration for 18 h. Lysates were subjected to immunoblotting analysis with the SRSF antibodies. AMPK and ACC were analyzed as controls. **b** A549 cells were glucose-starved for 12 h and then stimulated with glucose (25 mM) for the indicated times. Cell lysates were subjected to immunoblotting. **c** A549 cells maintained in 25 or 2.5 mM glucose were treated with cycloheximide (10 μg/ml) for the indicated times. SRSF5 protein level was analyzed by immunoblotting. **d** A549 cells were maintained at indicated concentration of glucose. Cells were harvested for ubiquitylation analysis. A549 cells (**e**) and H358 cells (**f**) were transfected with shRNA-SRSF5 or random shRNA by a lentivirus system. Cell numbers were determined by a cell counter at indicated times (upper panel) and the expression level of SRSF5 was determined by immunoblotting (lower panel). (**P < 0.01, two-way ANOVA test). **g** Tumor growth curves in nude mice. The indicated stable cell lines were collected and subcutaneously injected into nude mice. Tumor diameters were measured twice a week and tumor volume were calculated. Each point represents the mean volume ± s.e.m., *n* = 6 mice per group (**P < 0.01, Mann–Whitney test). **h** Tumor weight. All the tumors derived from indicated cells were shown and tumor weight was measured. Results are shown as mean ± s.e.m. of tumor weights (*n* = 6, each with initial six injections) (**P < 0.01, Mann–Whitney test). **i** Tumors shown in **h** were formalin-fixed, paraffin-embedded, and sliced for TUNEL assay. Representative images of indicated TUNEL staining are shown. The boxed areas in the right images were magnified on the left. Scale bar, 50 μm. Quantification of positive signals of TUNEL (**j**) or Ki67 (**k**) from indicated groups based on *n* = 100 cells assessed from six fields in **h** were shown. (*P < 0.05, one-way ANOVA test). Data are representative of three independent biological replicates (**e**, **f**; mean and s.e.m., *n* = 3). Unprocessed original scans of blots are shown in Supplementary Fig. 9

To investigate whether certain splicing factors respond to glucose intake, we screened all 12 members of SRSF family and examined their expression levels in A549 cells supplemented with different concentrations of glucose. Strikingly, the protein levels of SRSF5 were correlated with the concentration of glucose (Fig. 1a). SRSF3 expression displayed similar pattern with slower migration in the high glucose, suggesting a possible modification, which needs further verification. Other SRSFs kept on constant levels (Fig. 1a). The glucose fluctuation had no significant effects on SRSF5 mRNA levels, whereas the mRNA level of SRSF3 was dramatically up-regulated by glucose (Supplementary Fig. 1a, b). Similar results were observed in breast cancer MCF7 and hepatocellular cancer SMMC-7721 cells (Supplementary Fig. 1c, d). When glucose was re-introduced to glucose-deprived cells, the expression level of SRSF5 was markedly increased (Fig. 1b and Supplementary Fig. 1e, f). The half-life of SRSF5 was prolonged when the cells were under higher glucose (Fig. 1c). Low glucose robustly induced the ubiquitylation of SRSF5 (Fig. 1d). In this study, we focus on the function and regulation of SRSF5.

Since high glucose is necessary for rapid growth of tumor cells, we asked whether SRSF5 is involved in the tumorigenesis. Depletion of *SRSF5* in A549 and H358 lung cancer cells dramatically suppressed cell proliferation and tumor growth (Fig. 1e–h). SRSF5 knockdown also induced cell apoptosis, as revealed by TUNEL staining (Fig. 1i, j). Ki67 staining of xenografts showed that cell proliferation was also dramatically decreased (Fig. 1k). These results suggest that SRSF5 is stabilized by high glucose and functions as a tumor-promoting factor.

### SRSF5 controls CCAR1 splicing to regulate cell growth

**Fig. 2:**
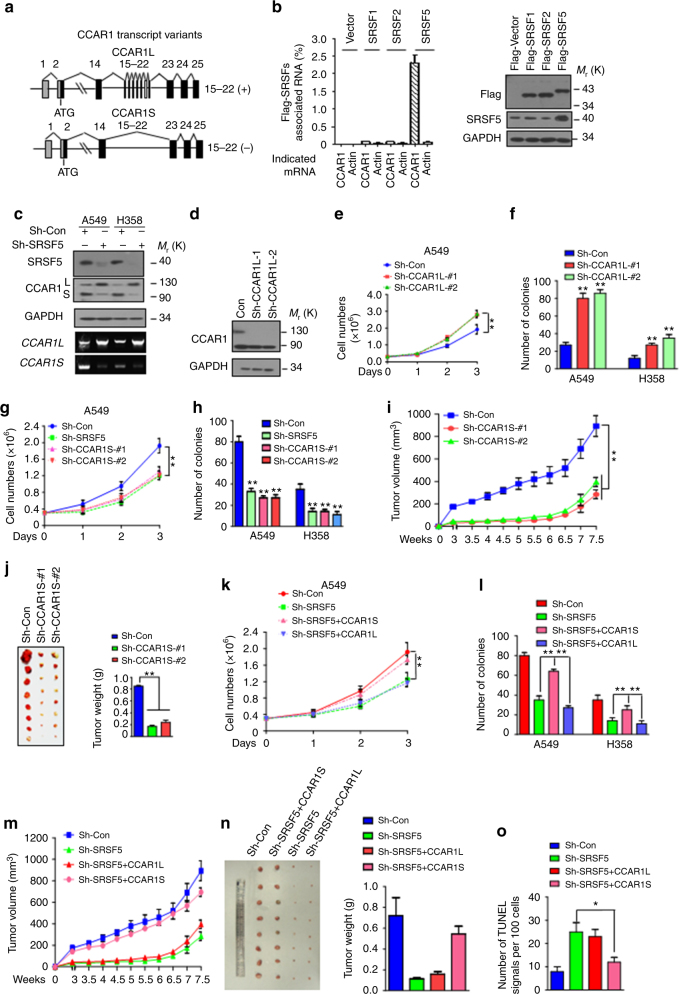
SRSF5 controls CCAR1 splicing to regulate tumor cell growth. **a** Schematic diagram of CCAR1 splice variants. **b** In vivo ultraviolet cross-linking and immunoprecipitation (CLIP) from cells transfected with indicated plasmids were subjected to qPCR analysis with specific primers (left panel). Expression of each protein was confirmed by immunoblotting (right panel). **c** Depletion of SRSF5 leads to a shift of CCAR1 splicing module in A549 and H358 cells. **d** CCAR1L knockdown efficiency in A549 cells was assessed by immunoblotting analysis. **e** Cell proliferation assay of cells as in **d** (**P < 0.01, two-way ANOVA test). **f** Quantification of clonogenic formation assay of indicated cells. **g** Reduced proliferation in the absence of CCAR1S in A549 cells. Cell proliferation assay was performed as in **e**, cells stably expressing shRNA-SRSF5 was introduced for comparison. **h** Reduced clonogenic formation ability in the absence of CCAR1S in A549 and H358 cells. **i** CCAR1S depletion delays tumor growth in nude mice. Tumor diameters were measured at indicated time points to calculate tumor volume. Each point represents the mean volume ± s.e.m. (**P < 0.01, Mann–Whitney test). **j** CCAR1S depletion shrinks tumor growth in nude mice. All the tumors were shown (left) and measured (right). Results are shown as mean ± s.e.m. of tumor weights (**P < 0.01, Mann–Whitney test). **k** Cell proliferation assay were conducted in A549/sh-SRSF5 cells re-introduced with CCAR1L, CCAR1S, and control vectors. **l** Quantification of the number of colonies for A549 cells or H358 cells. **m** CCAR1S re-introduction in *SRSF5*-depleted cells enhanced tumor growth in nude mice. Data were collected and displayed as in **i** (*P < 0.05, Mann–Whitney test). **n** CCAR1S re-introduction in *SRSF5*-depleted cells displays enhanced tumor weight. Data were collected and displayed as in **j** (**P < 0.01, two-way ANOVA test). **o** Quantification of the TUNEL signals of tumor sections in **n** is shown. *n* = 100 cells from three fields were assessed (*P < 0.05, one-way ANOVA test). Data are representative of three independent biological replicates (**e**–**h**, **k**, **l**; mean and s.e.m., *n* = 3). Unprocessed original scans of blots are shown in Supplementary Fig. 9

We next investigated the mechanism by which SRSF5 controls tumor growth. Given that SRSF5 is a classical splicing factor, we sought to identify its splicing target by RNA-seq. We found that exon skipping (10,173 events) was the most frequent in SRSF5 knockdown vs. control cells (Supplementary Fig. 2a). Four-way Venn diagrams illustrated a subset of overlapping genes between four types of AS in SRSF5 knockdown vs. control cells (Supplementary Fig. 2b). Among which the exon skipping of cell cycle and apoptosis regulator 1 (CCAR1) has been suggested as a possible target of SRSF5, although the physiological relevance remain unclear^19^. The long isoform of human CCAR1 has 25 exons (encoding CCAR1L, 1150 aa), while the short isoform is lack of exons 15–22 (encoding CCAR1S, 762 aa) (Fig. 2a and Supplementary Fig. 2c). To further measure whether SRSF5 binds CCAR1 mRNA in vivo, we performed cross-linked immunoprecipitation (CLIP) assays and found that SRSF5, but not SRSF1 and SRSF2, specifically bound to CCAR1 RNA (Fig. 2b). Strikingly, a dramatic S-to-L isoform switch was observed when SRSF5 was depleted (Fig. 2c). Interestingly, specific depletion of CCAR1L displayed increased growth rate and colony-formation capacity (Fig. 2d–f and Supplementary Fig. 2d, e). Stably overexpressing CCAR1L increased apoptosis, reduced colony-forming efficiency, and reduced tumor formation (Supplementary Fig. 2f–i). In contrast, stable depletion of CCAR1S inhibited cell growth, reduced colony formation and the tumor growth of xenograft, and increased apoptosis (Fig. 2g–j and Supplementary Fig. 2j–m). Overexpression of CCAR1S increased proliferation and promoted colonic formation (Supplementary Fig. 2n, o). These data indicate the tumor-suppressing and tumor-promoting role of CCAR1L and CCAR1S, respectively.

To test whether SRSF5 promotes tumor growth through CCAR1 splicing, CCAR1L or CCAR1S were stably introduced into SRSF5-depleted cells. The reintroduction of CCAR1S significantly increased cell proliferation and colony formation, while CCAR1L had no obvious effects (Fig. 2k, l and Supplementary Fig. 2p). Xenograft assays showed that overexpression of CCAR1S, but not CCAR1L, rescued the effects of SRSF5-depletion and promoted the tumor development (Fig. 2m–o). Furthermore, overexpression of anti-apoptotic Mcl-1S, another splicing target of SRSF5 in breast cancers^20^, did not rescue the effects of SRSF5 knockdown (Supplementary Fig. 2q–s). We also depleted three additional SRSFs, which have been reported to play fundamental roles in embryonic development and tissue homeostasis to determine the tumor formation capacity in vitro and in vivo. Neither of the depletion has suppressing effect (Supplementary Fig. 3a–d), indicating the specific role of SRSF5–CCAR1 axis in lung cancer.

### Network analysis of potential CCAR1L/S-associated proteins

**Fig. 3:**
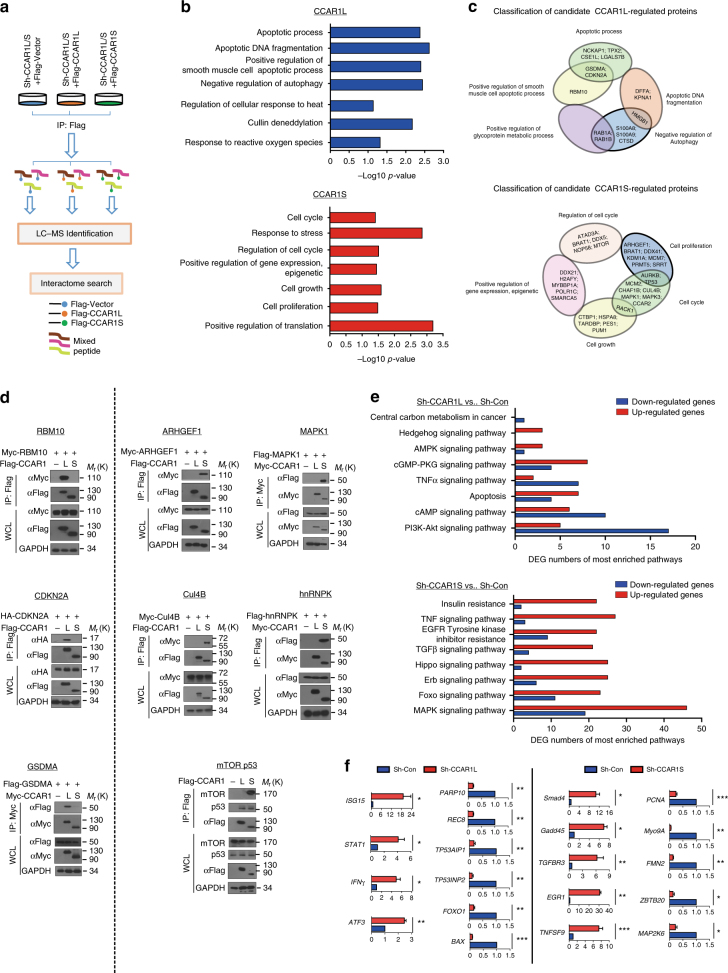
Network analysis of potential CCAR1L/S-associated proteins. **a** Experimental setup for the interactome analysis of the two CCAR1 isoforms (including CAR1L and CCAR1S). Equal amount of vector (control)-, CCAR1L-, or CCAR1S-overexpressing A549 cells were subjected to immunoprecipitation experiments using anti-Flag antibody followed by LC-MS/MS analysis. **b** Gene Ontology (GO) enrichment analysis for biological process, cell component, and molecular function of up-regulated proteins. The −log10 *P* value of enrichment is shown on *x* axis; the numbers represent the number of associated proteins for each term. **c** Classification of candidate CCAR1L and CCAR1S interacting proteins, which are thought to be specifically regulated by distinct isoforms. **d** IP-western validation of candidate CCAR1L (left) and CCAR1S (right) interacting proteins. CCAR1L, CCAR1S, and the indicated plasmids were transfected into A549 cells and co-immunoprecipitation assays were performed with the indicated antibodies followed by immunoblotting analysis. IP, immunoprecipitation; WCL, whole cell lysate. **e** KEGG analysis for differentially expressed genes (DEGs) among Sh-CCAR1L vs. Sh-Con, Sh-CCAR1S vs. Sh-Con groups. DEGs were identified following the criteria of log2ratio ≥ 1 and FDR ≤ 0.001. **f** Quantitative RT-PCR analysis of selected genes associated with enriched signaling pathways in CCAR1L- or CCAR1S-depleted A549 cells. Data are representative of three independent biological replicates. Unprocessed original scans of blots are shown in Supplementary Fig. 9

We next attempted to explore the mechanisms underlying the divergence of CCAR1L and CCAR1S observed in the cellular effects. Firstly, A549 cells restored with either CCAR1L or CCAR1S expression under CCAR1L/S depletion background were subjected to IP-MS analysis (Fig. 3a and Supplementary Fig. 3e, f). A total of 340 and 147 (for CCAR1L and CCAR1S, respectively) unique proteins were identified with the detection of at least one unique peptide at 1% false discovery rate (FDR), and those unique proteins were further used for subsequent GO term enrichment analysis in DAVID (Supplementary Data 1). Notably, in GO terms regarding biological process, proteins were mainly annotated as involved in apoptosis regulation, negative regulation of autophagy, and cullin deneddylation in CCAR1L interactome (Fig. 3b, upper panel), whereas cell cycle regulation, cell growth, and cell proliferation regulation as well as positive regulation of protein translation are the major GO terms in CCAR1S interactome (Fig. 3b, lower panel). Further, comparative analysis showed that a large number of candidate CCAR1L-specific proteins, such as CDKN2A, GSDMA, and RBM10, are involved in the regulation of cell apoptosis pathway. Also, many of the proteins are associated with negative regulation of autophagy, such as RAB1A, RAB1B, and HMGB1. These results are consistent with the definition of CCAR1 as cell cycle and apoptosis regulator 1 (Fig. 3c, upper). On the other hand, most of the candidate CCAR1S-specific proteins are associated with positive regulation of cell growth such as CTBP1 and PUM1, cell proliferation such as ARHGEF1 and DDX41, as well as cell cycle progression such as RACK1 and AURKB. In addition, we found that CCAR1S might play a role in the regulation of MAPK cascade and energy homeostasis, probably through association with certain components in the intracellular MAPK pathway such as MAPK1, MAPK3, mTOR (Fig. 3c, lower). We next carried out IP-western assays to validate a subset of the candidate-associated proteins based on the above bio-informatic analysis. As shown in Fig. 3d, RBM10, CDKN2A, and GSDMA were confirmed to interact with CCAR1L, but not with CCAR1S, in A549 cells (left panels), whereas ARHGEF1, Cullin 4B, MAPK1, mTOR, and hnRNPK were demonstrated to interact with CCAR1S, but not with CCAR1L (right panels). As a control, both CCAR1L and CCAR1S were shown to interact with p53 under these conditions (Fig. 3d, right). This result is consistent with a previous report showing that CCAR1 through its C-terminal coiled-coil domain interacts with p53 and serves as a co-activator of p53^21^.

We also performed RNA-seq in CCAR1L- or CCAR1S-depleted cells to unravel the distinct roles of CCAR1S and CCAR1L. A total of 694 differentially expressed genes (DEGs) were detected in Sh-CCAR1L vs. control cells (45.68% up-regulated, 54.32% down-regulated). On the other hand, a total of 2501 DEGs (31.19% up-regulated, 68.81% down-regulated) were detected in Sh-CCAR1S vs. control cells (Supplementary Fig. 3g, h and Supplementary Data 2). Detailed GO and KEGG pathway analysis demonstrated the divergences in distributions of multiple biological processes, molecular functions, and signaling pathways of these two isoforms (Fig. 3d, e and Supplementary Fig. 3i). Validation of the RNA-seq data by qRT-PCR showed that depletion of CCAR1L enhanced the expression of *ISG15*, *STAT1*, and *ATF3* but reduced the expression of *PARP10*, *TP53AIP1*, and *BAX*. Depletion of CCAR1S increased the expression of *GADD45*, *TNFSF9*, and *Smad4* but decreased the expression of *PCNA*, *Myo9A*, and *MAP2K6* (Fig. 3f). Collectively, these results help to understand why CCAR1L and CCAR1S isoforms execute such diverse functions in cell fate control.

### SRSF5 regulates glucose metabolism and acetyl-CoA production

**Fig. 4:**
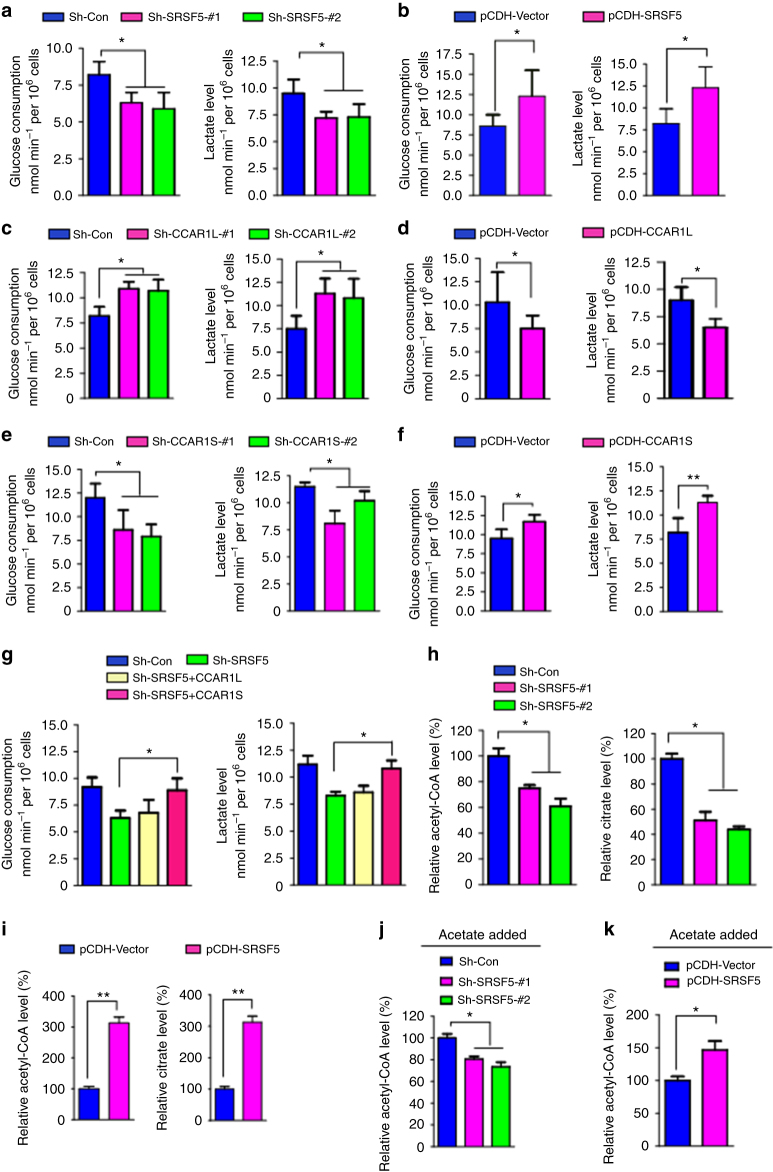
SRSF5 regulates glucose metabolism and acetyl-CoA production. **a** Analysis of glucose consumption rate and lactate production levels in SRSF5-depleted and control A549 cells. **b** Analysis of glucose consumption rate and lactate production levels in SRSF5-overexpressed and control A549 cells. **c** Analysis of glucose consumption rate and lactate production levels in CCAR1L-depleted and control A549 cells. **d** Analysis of glucose consumption rate and lactate production levels in CCAR1L-overexpressed cells. **e** Analysis of glucose consumption rate and lactate production levels in CCAR1S-depleted and control cells. **f** Analysis of glucose consumption rate and lactate production levels in CCAR1S-overexpressed cells. **g** Measurement of glucose consumption rate and lactate production levels in the cells described in Fig. 2k. **h** Acetyl-CoA levels and citrate levels in SRSF5-depleted and control A549 cells in high glucose. **i** Acetyl-CoA levels and citrate levels in SRSF5-overexpressed and control cells. **j** A549 cells depleted of SRSF5 were administrated to 5 mM acetate treatment and the relative acetyl-CoA level and citrate level were monitored. **k** A549 cells over-expressing SRSF5 were administrated to 5 mM acetate treatment and the relative acetyl-CoA level and citrate level were monitored. Data are representative of three independent biological replicates. All data are mean and s.e.m., *n* = 3. *P < 0.05 and **P < 0.01 (one-way ANOVA test)

During glycolysis, glucose is broken down into two three-carbon molecules of pyruvate and then converted into lactate. Since high abundance of SRSF5 positively correlates with high concentration of glucose, we set out to unravel the functional relationship between SRSF5 and glucose consumption. Interestingly, knockdown of SRSF5 led to a decrease in glucose consumption and lowered lactate production (Fig. 4a). Accordingly, stable expression of SRSF5 enhanced the glucose consumption and lactate production (Fig. 4b and Supplementary Fig. 4a). We also tested whether CCAR1 also regulates the glucose metabolism. Strikingly, knockdown of CCAR1L promoted while stable expression of CCAR1L suppressed glucose consumption and lactate production (Fig. 4c, d). Conversely, depletion of CCAR1S inhibited while CCAR1S overexpression enhanced these processes (Fig. 4e, f). Rescue experiment showed that CCAR1S but not CCAR1L restored the effect of SRSF5 depletion on glucose consumption and lactate production (Fig. 4g).

In cancer cells, glucose typically needs to be metabolized through the mitochondria to produce citrate in order to generate acetyl-CoA from ACL when nutrients are sufficient. Furthermore, acetate was recently found to be an alternative carbon source to synthesize acetyl-CoA by ACSSs when nutrients are avid^22^. Emerging evidence reveals that cells monitor the levels of acetyl-CoA as a key indicator of their metabolic state^23^, through distinctive protein acetylation modifications dependent on this metabolite. We examined the citrate level and acetyl-CoA level in SRSF5-depleted or overexpressed cells. As shown, SRSF5 depletion downregulated citrate and acetyl-CoA levels while SRSF5 overexpression upregulated those levels (Fig. 4h, i). These effects were reproduced in cells treated with acetate (Fig. 4j, k). Collectively, SRSF5 is involved in the regulation of glucose metabolism and acetyl-CoA production.

The tumor microenvironment is characterized by oxygen depletion, high lactate, and extracellular acidosis (lactic acidosis) as well as glucose deprivation. We have demonstrated that SRSF5 was downregulated by glucose starvation. In order to examine whether other environmental factor such as acidosis influences SRSF5, we detected SRSF5 level in the cells treated with lactic acid. Intriguingly, acidosis administration decreased SRSF5 protein level, accompanied by the increase of phosphorylated AMPK levels (Supplementary Fig. 4b, c). These results suggest that SRSF5 is regulated by multiple micro-environmental factors.

### Tip60 acetylates SRSF5 under high glucose

**Fig. 5:**
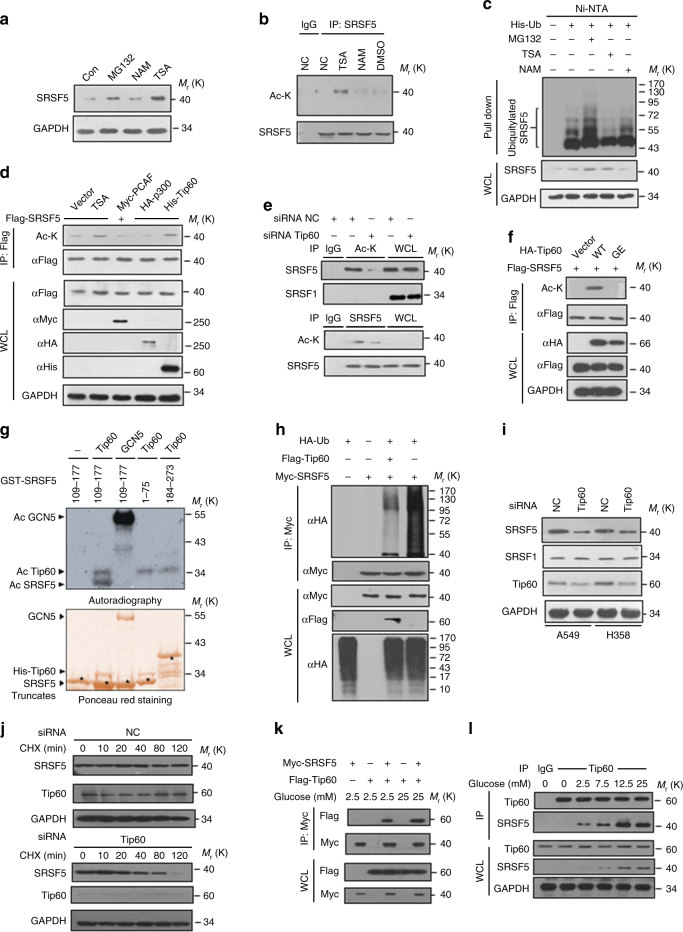
Tip60 acetylates SRSF5 under high glucose. **a** TSA, but not NAM, increases SRSF5 protein level. A549 cells were treated with or without NAM and TSA. Protein level of SRSF5 was measured by immunoblotting and MG132 treatment was used as a positive control. **b** Endogenous SRSF5 is acetylated. Endogenous SRSF5 protein was purified from HEK293T cells after NAM and TSA treatment as indicated. Acetylation levels were analyzed by immunoblotting. **c** In vivo competition between ubiquitylation and acetylation of SRSF5 were revealed by Ni^2+^ pull-down assay. **d** Overexpression of Tip60, but not other typical members of acetyltransferases, increases endogenous SRSF5 acetylation at K125. **e** *Tip60* knockdown sharply reduced SRSF5 acetylation level. **f** Acetyltransferase-activity of Tip60 is required for SRSF5 K125 acetylation. Flag-tagged SRSF5 was co-transfected with HA-tagged Tip60 WT or catalytically inactive mutant G380E into HEK293T cells. Acetylation was determined by immunoblotting. **g** Purified GST–SRSF5 fusion proteins were incubated with recombinant His-tagged Tip60^212–513^ or hGCN5 in the presence of [^14^C] acetyl-CoA. Acetylation was revealed after autoradiography (upper panel). Equivalent amounts of various recombinant proteins were assessed by Ponceau red staining (lower panel). **h** Overexpression of Tip60 decreases SRSF5 ubiquitylation. Flag-tagged Tip60 was co-transfected with Myc-tagged SRSF5 and HA-tagged ubiquitin. The ubiquitylation of SRSF5 was determined by IP-Western with anti-HA antibody. **i** Depletion of Tip60 decreases SRSF5 protein level in A549 and H358 cells. **j** *Tip60* knockdown decreases SRSF5 protein stability. A549 cells were transfected with si*Tip60* or control were treated with CHX as previously described. The endogenous SRSF5 protein was determined and quantified by immunoblotting against GAPDH. **k** A549 cells were transfected with Myc-SRSF5 and Flag-Tip60 as indicated and the cells were treated with glucose of either 2.5 or 25 mM. Co-immunoprecipitation assays were performed to indicate the interaction between Tip60 and SRSF5. **l** Low glucose decreases the physiological interaction between SRSF5 and Tip60. A549 cells were treated with glucose of indicated concentrations and co-immunoprecipitation assays were performed to determine the dynamic interactions of Tip60 and SRSF5. Data are representative of three independent biological replicates. Unprocessed original scans of blots are shown in Supplementary Fig. 9

We next investigated the mechanisms of how SRSF5 proteins are maintained under high glucose. Several independent proteomics studies have revealed large numbers of acetylation proteins involved in nuclear processes including RNA splicing^24,25^, among which, the SRSF5 acetylation was documented. To verify this event, we firstly analyzed the protein level of SRSF5 upon treatment with different types of histone deacetylase (HDAC) inhibitors. SRSF5 was significantly increased upon treatment with trichostatin A (TSA), an inhibitor of HDAC class I and class II (Fig. 5a and Supplementary Fig. 5a, b). The mRNA level of SRSF5 displayed minimal fluctuations (Supplementary Fig. 5c), confirming the change occurred at post-transcriptional level. Importantly, both transfected and endogenous SRSF5 were detected to be acetylated when the cells were treated with TSA (Fig. 5b and Supplementary Fig. 5d). Furthermore, TSA treatment inhibited the ubiquitylation of SRSF5 (Fig. 5c).

To identify the acetyltransferase responsible for SRSF5, we examined three representative acetyltransferases, and observed that Tip60 (TAT-interacting protein, 60 kDa) specifically promoted SRSF5 acetylation (Fig. 5d). When endogenous Tip60 was depleted (Supplementary Fig. 5e), the acetylation of SRSF5 was dramatically decreased (Fig. 5e). Tip60-WT, but not its catalytic-inactive mutant G380E, promoted the SRSF5 acetylation in cells (Fig. 5f). *R*ecombinant Tip60 was able to efficiently acetylate SRSF5 in vitro and the acetylation site was located within the region 109–177 encompassing the RRMH domain. The effect was specific to Tip60 since GCN5 was unable to acetylate SRSF5 (Fig. 5g and Supplementary Fig. 5f). Furthermore, the ubiquitylation level of SRSF5 declined when Tip60 was overexpressed (Fig. 5h). Conversely, when Tip60 was depleted, the half-life of SRSF5 was shortened and the protein level of SRSF5 declined (Fig. 5i, j). Consistently, we could readily detect the interaction between SRSF5 and Tip60, and high glucose enhanced their interaction (Fig. 5k, l and Supplementary Fig. 5g, h). These results suggest that Tip60 plays a major role in promoting SRSF5 acetylation and maintaining SRSF5 stability.

### Smurf1 targets SRSF5 for degradation upon low glucose

**Fig. 6:**
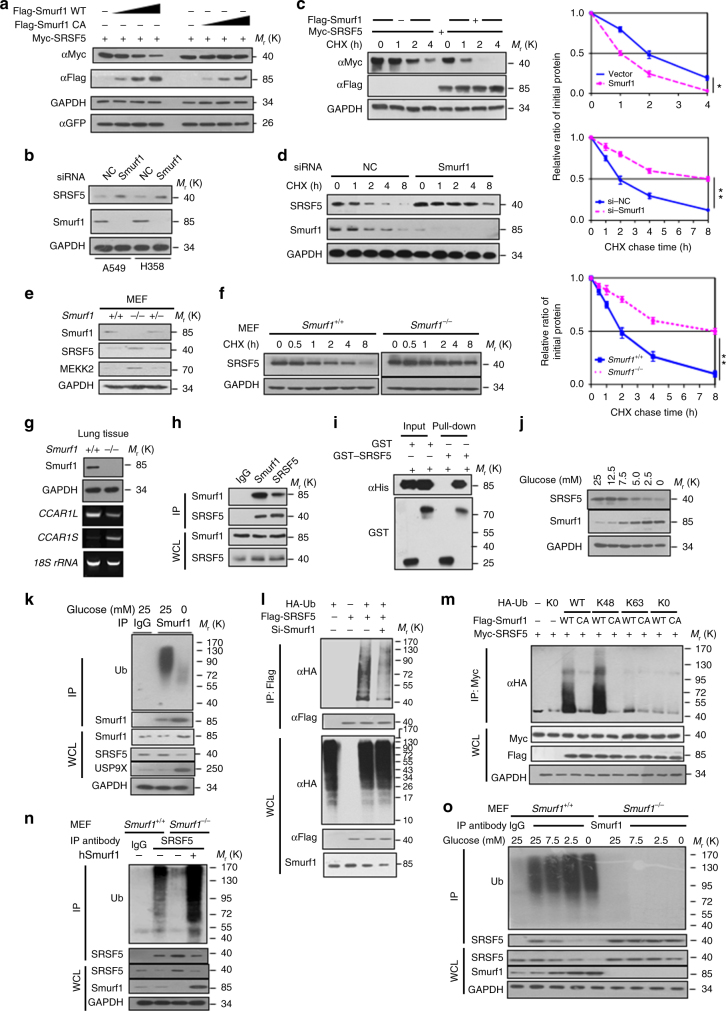
Smurf1 targets SRSF5 for degradation upon low glucose intake. **a** Smurf1 negatively regulates SRSF5 protein level in a dose-dependent manner. HEK293T cells were transfected with increasing amounts of Flag–Smurf1 WT and or Flag–Smurf1 CA vectors along with Myc-SRSF5 vectors for immunoblotting analysis. **b** *Smurf1* knockdown increases the expression level of SRSF5 in A549 and H358 cells. **c** HEK293T cells transfected with the indicated plasmids were treated with CHX and harvested at the indicated times for western blot. **d** A549 cells transfected with si*Smurf1* or control as previously described were treated with CHX for the indicated time to determine endogenous SRSF5 expression levels. **e** Expression analysis of endogenous SRSF5 protein in *Smurf1*^+/+^, *Smurf1*^+/−^, and *Smurf1*^−/−^ MEFs were revealed by immunoblotting. **f** Half-life analysis of SRSF5 in *Smurf1*^+/+^ and *Smurf1*^−/−^ MEFs. **g** Expression levels of CCAR1 splice variants were examined in the lung tissues of *Smurf1*^+/+^ and *Smurf1*^−/−^ mice by RT-PCR. **h** Co-immunoprecipitation assay revealed that endogenous SRSF5 interacts with Smurf1 in A549 cells. **i** GST pull-down assays were performed to indicate the direct interaction between Smurf1 and SRSF5. **j** The expression level of Smurf1 reversely correlates with SRSF5 when glucose concentration declines. **k** Glucose deprivation dampens endogenous Smurf1 ubiquitylation. A549 cells were maintained in medium with or without 25 mM glucose. The endogenous Smurf1 ubiquitylation level were determined by IP-western. **l** Knockdown of *Smurf1* decreases SRSF5 ubiquitylation. HEK293T cells with or without si*Smurf1* were transfected with indicated plasmids. The ubiquitylation of SRSF5 was determined by IB analysis. **m** Smurf1 promotes the K48-linked poly-ubiquitylation of SRSF5 in vivo. The SRSF5 ubiquitylation linkage was analyzed in HEK293T cells transfected with indicated plasmids. **n** Ectopic expression of hSmurf1 by lentiviral infection rescues the ubiquitylation of endogenous SRSF5 in *Smurf1*^−/−^ MEFs. **o** Smurf1 ubiquitylates SRSF5 by sensing glucose concentration. *Smurf1*^+/+^ and *Smurf1*^−/−^ MEFs were maintained under various glucose concentrations and ubiquitylated SRSF5 was visualized. Blots are representative of three independent biological replicates. Error bars in **c**, **d**, **f** show s.e.m. from three independent experiments (**P < 0.01, two-way ANOVA test). Unprocessed original scans of blots are shown in Supplementary Fig. 9

The HECT-type E3 Smurf1 (Smad ubiquitylation regulatory factor 1) has been identified as a putative SRSF5-interacting protein in a high-throughput analysis of TGF-β signaling network^26^. This evidence prompted us to examine whether Smurf1 is a potential E3 ligase for SRSF5. Ectopic expression of Smurf1, but not its catalytic-inactive mutant C699A, or other members of Smurf1/Nedd4 family, resulted in proteasomal degradation of SRSF5 (Fig. 6a and Supplementary Fig. 6a, b). Depletion of Smurf1 upregulated SRSF5 protein level while the mRNA level of SRSF5 was largely unchanged (Fig. 6b and Supplementary Fig. 6c). Consistently, the half-life of SRSF5 was shortened by overexpression of Smurf1, and prolonged by depletion of Smurf1 (Fig. 6c, d). To prove that Smurf1 affects SRSF5 in vivo, we generated *Smurf1* knockout mice and found that SRSF5 protein level was upregulated in *Smurf1*-deficient mouse embryonic fibroblasts (MEFs) and multiple adult tissues, and the half-life of SRSF5 protein was significantly prolonged in *Smurf1*^−/−^ MEFs (Fig. 6e, f and Supplementary Fig. 6d). The SRSF5 mRNA level remained unchanged (Supplementary Fig. 6e). Exclusion of CCAR1 exons 15–22 was dramatically increased in *Smurf1*^−/−^ lung tissue compared with that in the WT littermates (Fig. 6g). Binding assays showed that SRSF5 interacted with Smurf1 both in vivo and in vitro (Fig. 6h, i and Supplementary Fig. 6f). The HECT domain of Smurf1 and the RS (arginine–serine) domain of SRSF5 mediated this interaction (Supplementary Fig. 6g–i). Importantly, we observed gradually decreased SRSF5 and increased Smurf1 concurrent with decreasing concentrations of glucose (Fig. 6j). This striking effect led us to consider the mechanisms underlying the induction of Smurf1 during glucose deprivation. The mRNA level of Smurf1 remained comparable when glucose fluctuate in concentration or time manners (Supplementary Fig. 6j, k). Instead, the ubiquitylation level of Smurf1 was sharply declined when the cells were deprived of glucose, accompanied by significantly elevated protein level of USP9x (Fig. 6k), a reported deubiquitylase of Smurf1^27^. These results suggest that the deubiquitylation of Smurf1 might contribute to its protein stabilization under low glucose.

Next, we examined the ubiquitylation of SRSF5 by Smurf1. The ubiquitylation of the transfected SRSF5 was easily detectable, and depletion of Smurf1 markedly attenuated the SRSF5 ubiquitylation in cells (Fig. 6l). Smurf1-WT, but not its C699A mutant, directly promoted the ubiquitylation on SRSF5 and the catalytic HECT domain of Smurf1 was both sufficient and required for the ubiquitylation of SRSF5 (Supplementary Fig. 6l, m). Notably, Smurf1 effectively promoted K48-type poly-ubiquitylation, but not mono-ubiquitylation, nor the non-degradative K63-type poly-ubiquitylation of SRSF5 (Fig. 6m). We also observed a sharp decrease of SRSF5 ubiquitylation level in *Smurf1*^−/−^ MEFs, and the reintroduction of human Smurf1 restored the ubiquitylation (Fig. 6n). Furthermore, glucose starvation induced SRSF5 ubiquitylation in Smurf1-WT MEFs, but not in *Smurf1*^−/−^ MEFs (Fig. 6o), confirming the dependence of Smurf1 for SRSF5 ubiquitylation. Taken together, these results strongly indicate that Smurf1 is a bona fide ubiquitin ligase for SRSF5.

### Acetylation of SRSF5 protects it from degradation

**Fig. 7:**
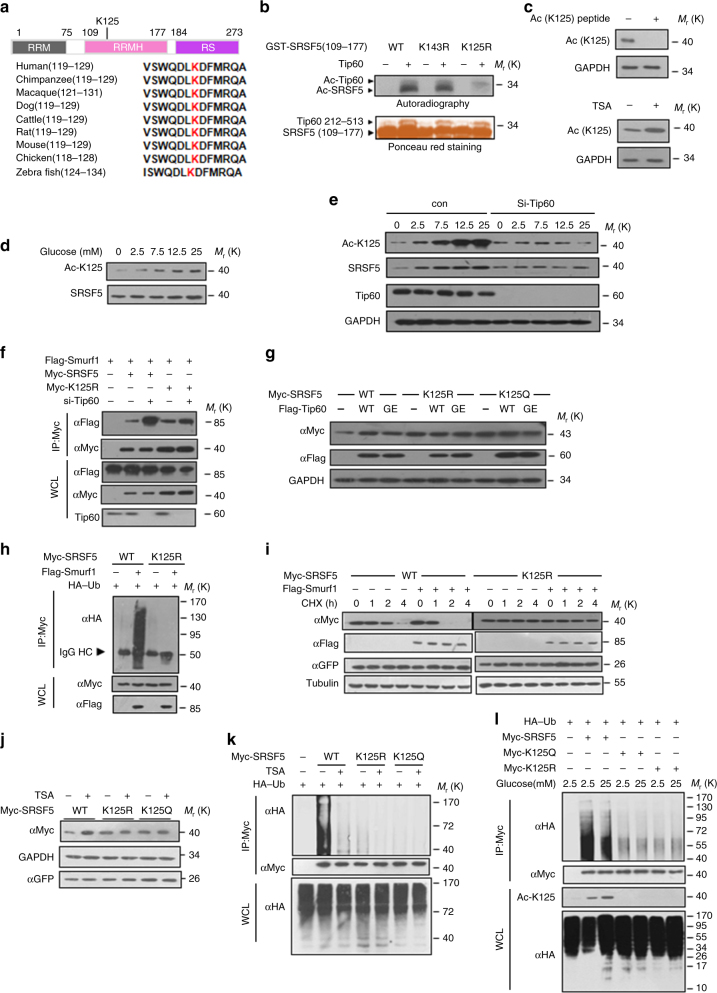
Acetylation of SRSF5 protects it from degradation. **a** Multiple sequences alignments of SRSF5 across species. **b** K125 is a prime-candidate site for Tip60-mediated acetylation as detected by autoradiography. **c** Confirmation of Ac-K125 antibody activity. **d** Glucose increases SRSF5 K125 acetylation level. The loading was normalized to SRSF5 protein levels so as to indicate the relative acetylation level. **e** Tip60 is required for glucose-regulated SRSF5 acetylation. Endogenous basic and acetylated level of SRSF5 in control and *Tip60* knockdown cells in response to different glucose concentration were detected by immunoblotting. **f** Inhibition of Tip60 increases the interaction between Smurf1 and wild-type SRSF5, but not K125R. HEK293T cells treated with or without Tip60 were transfected with indicated plasmids. The interaction between SRSF5 and Smurf1 was determined by IP-western. **g** Myc-tagged SRSF5 WT, K125R, K125Q plasmids were co-expressed for 36 h in A549 cells with either wild-type (WT) HA–Tip60 or mutant (G380E) HA–Tip60. Immunoblotting analysis using anti-Myc antibody is presented. **h** Amino acid K125 of SRSF5 is required for the ubiquitylation mediated by Smurf1 in vivo. HEK293T cells transfected with indicated plasmids were subjected to ubiquitylation analysis, as revealed by immunoblotting. **i** Substitution of SRSF5 lysine 125 to arginine prolongs its half-life. HEK293T cells were transfected with plasmids as indicated. Cells were subjected to CHX treatment for indicated times and the lysates were analyzed. **j** TSA treatment increases the abundance of SRSF5 WT but not K125 mutants. Myc-tagged SRSF5 K125 WT or mutant plasmids were transfected into HEK293T cells with or without TSA treatment. Expression of SRSF5 were analyzed by immunoblotting. **k** TSA decreases the ubiquitylation of SRSF5 WT, but not K125 mutants. HEK293T cells were transfected with indicated plasmids with or without TSA treatment. Ubiquitylation of purified proteins was analyzed. **l** High glucose decreases the ubiquitylation of SRSF5 WT, but not K125 mutants. HEK293T cells transfected with indicated plasmids were maintained under 2.5 or 25 mM glucose concentrations. Ubiquitylation and acetylation of purified proteins were analyzed. Data are representative of three independent biological replicates. Unprocessed original scans of blots are shown in Supplementary Fig. 9

A recent mass spectrometry-based proteomics study has identified a myriad of potentially ubiquitylated proteins including SRSF5^28^. Notably, among all classical SRSFs, SRSF5 is the only member whose ubiquitylation peptide was identified with high fidelity. The potential ubiquitylation site K125 received high scores^28^ and this lysine was evolutionary conserved through zebrafish to mammals (Fig. 7a). In vitro acetyltransferase assays showed that SRSF5 K125 site was also the major site of acetylation by Tip60 (Fig. 7b). To further confirm the acetylation on K125 of SRSF5, we generated an antibody specific to K125-acetylated SRSF5 protein. The specificity of the antibody was confirmed by its ability to recognize the acetylated, but not unacetylated, peptide (Supplementary Fig. 7a–d). The acetylation signal was blocked by pre-incubating the antibody with antigen peptides and enhanced by treatment with TSA (Fig. 7c). Using this antibody, we observed elevated acetylation level of endogenous SRSF5 along with increasing glucose concentration (Fig. 7d). Moreover, Tip60 depletion abolished the glucose-mediated upregulation of SRSF5 acetylation (Fig. 7e), indicating that high glucose-induced SRSF5 acetylation is dependent on Tip60. Interestingly, when Tip60 was depleted, the interaction between SRSF5 and Smurf1 was increased (Fig. 7f). However, Tip60 had only weak effect on the interaction between Smurf1 and SRSF5–K125R mutant (Fig. 7f). Tip60 stabilized SRSF5-WT, but not K125R (un-acetylated) or K125Q (acetylation mimicked) mutant (Fig. 7g). K125 was also the major site of SRSF5 ubiquitylation mediated by Smurf1 (Fig. 7h). K125R mutant was resistant to Smurf1-mediated degradation (Fig. 7i). The K125R and K125Q mutants were insensitive to TSA treatment (Fig. 7j, k and Supplementary Fig. 7e). Increased glucose concentration reduced the ubiquitylation level of SRSF5 dependent on K125 (Fig. 7l). Hence, we conclude that Tip60-mediated acetylation of SRSF5 on K125 protects it from Smurf1-mediated ubiquitylation and degradation.

### HDAC1 deacetylates SRSF5 upon low glucose

**Fig. 8:**
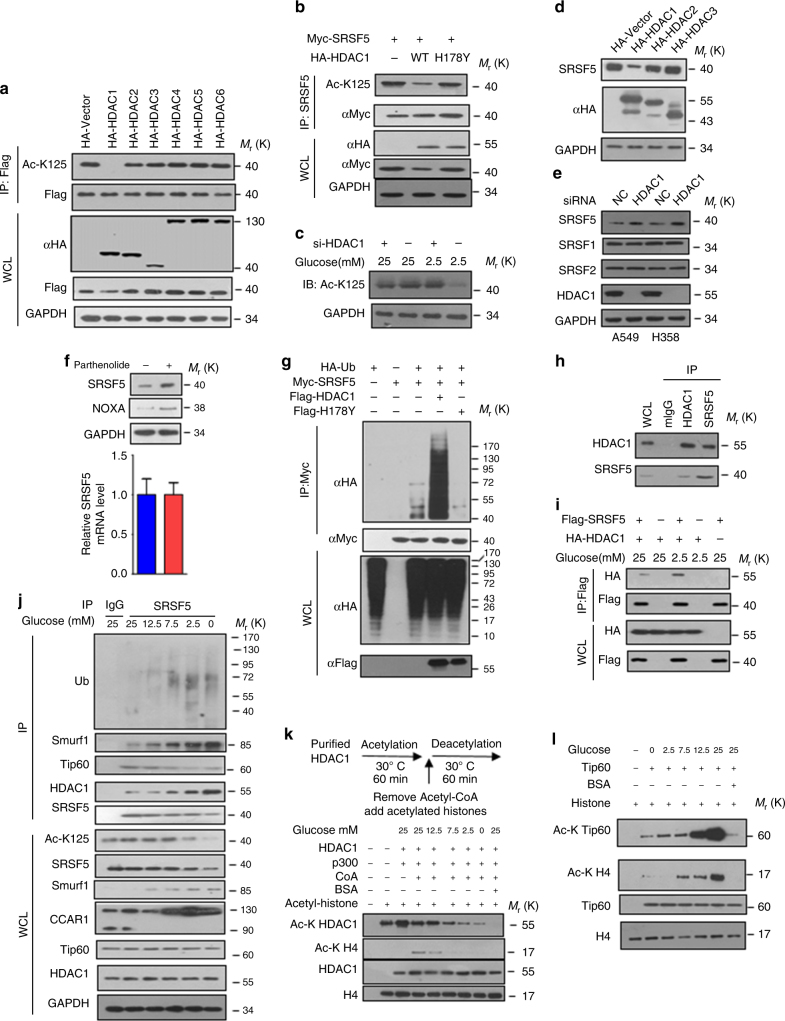
HDAC1 deacetylates SRSF5 upon low glucose. **a** HDAC1 overexpression specifically decreases SRSF5 acetylation. HEK293T cells were transfected with indicated plasmids and the acetylation levels of SRSF5 were determined by immunoblotting. **b** Catalytic activity of HDAC1 is required for the deacetylation of SRSF5. Myc-tagged SRSF5 was co-transfected with HA-tagged HDAC1 WT or catalytically inactive mutant H178Y into HEK293T cells. Acetylation was determined by immunoblotting. **c** HDAC1 is required for glucose-regulated SRSF5 acetylation. HEK293T cells transfected with or without siHDAC1 were cultured in medium containing 2.5 or 25 mM glucose. **d** Overexpression of HDAC1 specifically decreases SRSF5 protein level as revealed by immunoblot. **e** *HDAC1* knockdown increases SRSF5 protein level in A549 and H358 cells. **f** A549 cells were treated with or without HDAC1 inhibitor Parthenolide. Endogenous SRSF5 protein levels were determined by immunoblot analysis and relative SRSF5 mRNA levels were quantified by qPCR. **g** HDAC1, but not its catalytic-inactive mutant, promotes SRSF5 ubiquitylation. HEK293T cells transfected with Myc-tagged SRSF5, Flag-tagged HDAC1 WT or H178Y vectors, HA-tagged ubiquitin were subjected to ubiquitylation analysis, as revealed by immunoblotting. **h** Endogenous interaction between SRSF5 and HDAC1 were revealed by co-immunoprecipitation assays. **i** High glucose decreases the interaction between HDAC1 and SRSF5. Flag-tagged SRSF5 were co-transfected with HA-tagged HDAC1 into HEK293T cells upon different glucose concentrations. Protein interactions were determined. **j** A549 cells were maintained at various glucose concentrations for 18 h and harvested for immunoprecipitation and immunoblotting analysis. **k** HDAC1 activity is inhibited by acetylation at high concentration. HDAC1 purified from HEK293T cells maintained at different concentrations of glucose was first acetylated with p300. Acetyl-CoA was then removed by dialysis, and the samples were incubated with acetylated core histones to examine the HDAC1 deacetylase activity. **l** Glucose protrusion promoted Tip60 autoacetylation. Tip60 purified from A549 cells maintained at indicated glucose concentration was incubated with H4, [^3^H]-acetyl CoA, or BSA (1 mg) as indicated, and the auto-acetylation was detected by immunoblotting. Data are representative of three independent biological replicates (**f**; mean and s.e.m., *n* = 3). Unprocessed original scans of blots are shown in Supplementary Fig. 9

Recent studies have shown that acetylation of numerous proteins, such as PTEN^29^, PPARγ^30^, p53^31^, is a dynamic process that can be catalytically reversed by specific deacetylases. We sought to identify the specific HDAC member for SRSF5. Among the examined HDACs, only HDAC1 specifically deacetylated SRSF5 (Fig. 8a). Ectopic expression of HDAC1, but not its catalytic-inactive mutant H178Y, led to a significant decrease of SRSF5 acetylation on K125 (Fig. 8b). Depletion of HDAC1 increased the SRSF5 acetylation under low glucose (Fig. 8c). Co-expression of HDAC1 downregulated the SRSF5 protein level (Fig. 8d), while SRSF5 mRNA expression unchanged (Supplementary Fig. 8a). Depletion of HDAC1 or treatment with parthenolide, an HDAC1-specific inhibitor, resulted in elevated expression of SRSF5 protein (Fig. 8e, f) without significant effect on SRSF5 mRNA levels (Supplementary Fig. 8b). Furthermore, the ubiquitylation level of SRSF5 was increased when overexpressing HDAC1-WT, but not its H178Y mutant (Fig. 8g and Supplementary Fig. 8c). SRSF5 was readily co-immunoprecipitated with HDAC1 (Fig. 8h). The HDAC1 binding region was mapped to the RRMH domain of SRSF5 (Supplementary Fig. 8d). High glucose decreased the interactions between SRSF5 and HDAC1 (Fig. 8i). Additionally, when co-expressed with HDAC1, the acetylation level as well as protein stability of SRSF5 was sharply reduced (Supplementary Fig. 8e, f). Consistently, when knocking down HDAC1, the protein level of SRSF5-WT was dramatically increased while K125R or K125Q showed minor effects (Supplementary Fig. 8g).

Gradual reduction of glucose concentration resulted in decreased protein levels and increased ubiquitylation levels of SRSF5, accompanied by its increased associations with Smurf1 and HDAC1and dissociation from Tip60 (Fig. 8j). Accordingly, the splicing of CCAR1 diminished (Fig. 8j). Notably, the increased acetylation level of HDAC1 itself in high glucose impairs its deacetylase activity towards substrates (Fig. 8k), On the contrary, high glucose induced the acetyl-transferase activity of Tip60 through enhancing its cis-acetylation level (Fig. 8l). To sum up, these results support the notion that under low glucose, HDAC1 predominantly interacts with SRSF5 and maintains SRSF5 at low acetylation level, which allows SRSF5 ubiquitylation by Smurf1; whereas under high glucose, Tip60 predominantly interacts with SRSF5 and maintains SRSF5 at high acetylation level to protect SRSF5 from degradation.

### K125 mutants of SRSF5 promotes tumor growth

**Fig. 9:**
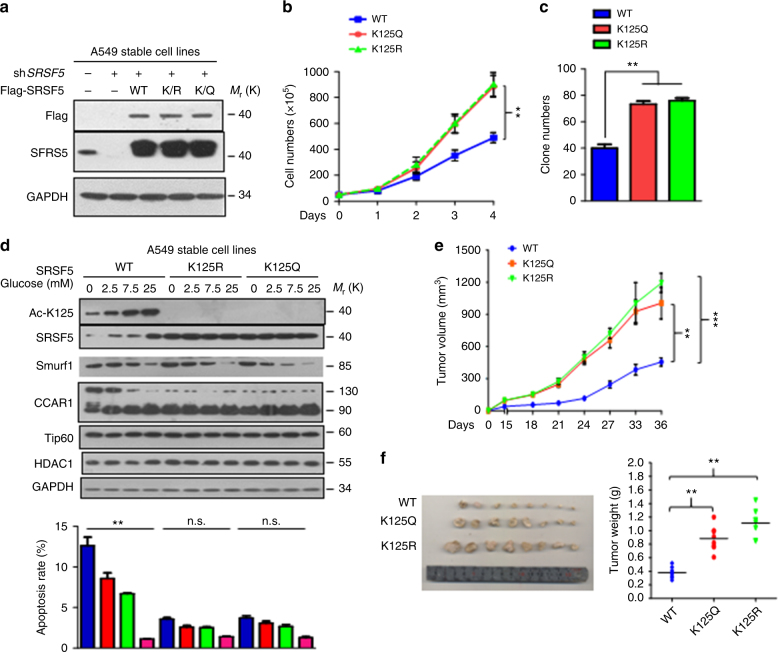
K125 mutants of SRSF5 promote tumor cell growth. **a** Verification of A549 stable cell lines. Knockdown efficiency and re-expression levels of wild-type or K125R/Q mutants were determined by immunoblotting. **b** K125R or K125Q A549 stable cell lines displayed higher proliferative rate than that of the WT cells. Cell numbers of indicated cell lines were counted every 24 h after seeding. Error bars represent cell numbers ± s.e.m. for triplicate experiments. The two-tailed Student’s *t*-test was used. **P < 0.01. **c** Quantification of the number of colonies for A549 cells as described in **b**. **d** The apoptosis ratio of A549 cells stably expressing WT, K125R, K125Q mutant subjected to various glucose concentration were determined (lower) and the indicated protein level of SRSF5, Smurf1, CCAR1 were determined by immunoblotting analysis (upper). (**P < 0.01, one-way ANOVA test). **e**, **f** K125R and K125Q mutants promote xenograft tumor growth. Subcutaneous xenograft experiment was performed in nude mice using A549 stable cells. Major and minor diameters of tumors were measured and tumor volumes were calculated. The two-tailed Student’s *t*-test was used. *P < 0.05; **P < 0.01; ***P < 0.001; NS denotes no significance (**e**). Thirty-six days after injection, tumors were dissected, photographed, and weighted. The two-tailed Student’s *t*-test was used. **P < 0.01; NS denotes no significance (**f**). Data are representative of three independent biological replicates (**d**; mean and s.e.m., *n* = 3). Unprocessed original scans of blots are shown in Supplementary Fig. 9

To provide more evidence that the modification of SRSF5 mediates the metabolic stress response of lung cancer cells, we established three A549 stable lines that knocked down endogenous SRSF5 and then further introduced SRSF5 WT or the K125R, K125Q mutants (Fig. 9a). The cells expressing K125R or K125Q mutant proliferated significantly faster than the cells expressing SRSF5-WT (Fig. 9b). High capacity of colony formation were also observed (Fig. 9c). We therefore determined the glucose sensing capacity between the SRSF5-WT and K125 mutant cells. Glucose induced acetylation and stabilization of SRSF5, decline of Smurf1, and switch of CCAR1 from L isoform to S isoform, accompanied by decreased cell apoptosis (Fig. 9d). Strikingly, cells expressing SRSF5 acetylation-deficient mutant K125R or acetylation-mimicking mutant K125Q were insensitive to glucose fluctuation although Smurf1 was still declined, resulting in constitutive splicing of CCAR1 and anti-apoptotic effects of the cells (Fig. 9d). Furthermore, xenograft experiments using these stable cell lines showed that the K125R and K125Q mutant markedly promoted tumor growth faster than the SRSF5-WT (Fig. 9e, f). These data highlight the significance of reciprocal shift of acetylation and ubiquitylation on SRSF5 K125 between glucose sufficient and insufficient conditions.

### SRSF5 status correlates with CCAR1 splicing and tumorigenesis

**Fig. 10:**
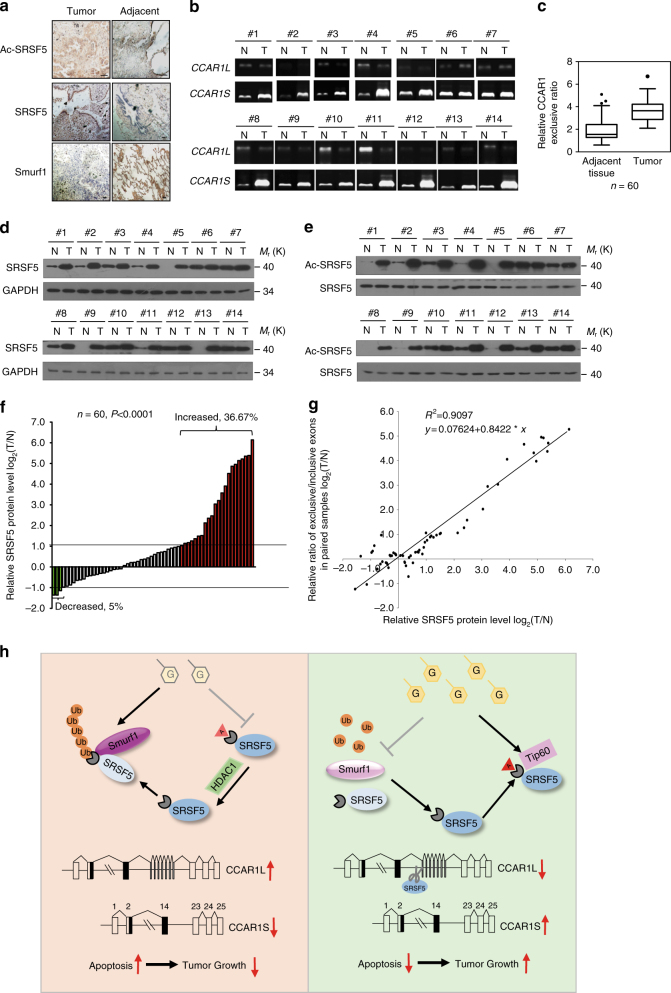
SRSF5 status correlates with CCAR1 splicing and tumorigenesis. **a** Representative images from immune-histochemical staining of Ac-SRSF5, SRSF5, and Smurf1 in three serial sections of the same tumor and matched adjacent tissue. Scale bar, 50 μm. **b** Total RNAs from 60 paired human NSCLC (T) and normal tissues (N) were examined by RT-PCR. Representative results for detection of CCAR1 exons 15–22 splicing patterns are shown. **c** Quantification of data from **b** for exons 15–22 exclusion ratio. The median box and whiskers plot was then calculated for the paired normal and tumor sets using Wilcoxon matched pairs test (*P < 0.05, one-way ANOVA test). **d** Lung cancer clinical cases with an increase in SRSF5 protein level. Human lung carcinoma samples paired with carcinoma tissue (shown as T) and adjacent normal tissue (shown as N) were lysed. The total SRSF5 protein levels were analyzed by immunoblotting analysis. **e** Lung cancer clinical cases with increased SRSF5 acetylation level at K125 in SRSF5-upregulated NSCLC. Human lung carcinoma samples paired with carcinoma tissue (shown as T) and adjacent normal tissue (shown as N) were lysed. The acetylated protein levels were compared against SRSF5 in immunoblotting analysis. **f** Relative expression of SRSF5 protein level in paired human clinical lung cancer samples and normal tissues. Immunoblotting analysis was performed on 60 paired human clinical lung cancer samples. Expression levels of SRSF5 were normalized to that of GAPDH. Data were calculated from triplicates. Bar value is the log ratio of SRSF5 expression levels between lung cancer samples (T) and matched normal tissues (N) from the same patient. Bar value ≤ −1 represents SRSF5 is decreased in tumors. Bar value > 1 represents that SRSF5 is increased in tumors. **g** Positive correlation between CCAR1 exclusive exons 15–22/inclusive exons 15–22 ratio and expression levels of SRSF5 was observed in human clinical lung samples. Relationships between these two variables were determined by Pearson’s correlation coefficients. **h** Model for ubiquitylation and acetylation of SRSF5 regulating alternative splicing of CCAR1 in signaling glucose sufficiency. Unprocessed original scans of blots are shown in Supplementary Fig. 9

We next asked whether expression of SRSF5 as well as acetylated SRSF5 is abnormally altered in clinical lung cancers. To this end, we examined SRSF5 expression levels in 60 pairs of human lung cancer samples and their matched normal lung epithelial tissues by immunohistochemical (IHC) analysis. The staining of total SRSF5 and acetylated SRSF5 increased, while the Smurf1 expression level decreased moderately, in lung cancer samples compared with their adjacent tissues and accumulation of positive signals in nucleus were observed (Fig. 10a). Analysis of CCAR1 mRNA showed that CCAR1S transcript increased in tumor tissues compared with normal tissues and representative RT–PCR results are shown in Fig. 10b. We also observed almost triple increased levels of exons 15–22 exclusion vs. inclusion in tumor samples compared to that of normal tissues (3.24 ± 0.86/1.36 ± 0.78, *n* = 60) (Fig. 10c). Approximately 2-fold increase of exclusive 15–22 exons in tumors vs. exclusive 15–22 in adjacent tissues was observed in 40% of the paired samples, and the largest change fold was 6.8 (Supplementary Table 3). Notably, increased exons 15–22 exclusion was probably associated with the higher tumor grade (*P* = 0.0119; Supplementary Table 1). These data suggest that the CCAR1S isoform plays a role in the development of human lung cancer.

Expression levels of SRSF5 were also analyzed by western blot and further shown as a ratio between SRSF5 and the reference gene GAPDH to correct for the variations. Upregulation of SRSF5 (2-fold) occurred in 22 of 60 (36.67%) clinical lung cancer samples compared with the paired normal tissues (Fig. 10d, f; red columns). Univariate analysis showed that protein levels of SRSF5 were significantly different between paired normal and cancer samples (*P* < 0.001, Fig. 10f). In addition, analysis of SRSF5 acetylation normalized with total SRSF5 showed similar upregulation (Fig. 10e). The increased expression of SRSF5 was associated with higher tumor grade (*P* = 0.0327; Supplementary Table 2). Additionally, a positive correlation (*R*^2^ = 0.9070) was observed between the ratio of SRSF5 expression level and the ratio of CCAR1 splice variants (exons 15–22 exclusion/inclusion) in paired clinical samples (Fig. 10g). Together, these results indicate that SRSF5 is involved in human lung cancer development, probably through regulating alterative splicing of CCAR1 pre-mRNA.

## Discussion

In this study, we demonstrate that the classical splicing factor SRSF5 plays a critical role in sensitizing the alteration of glucose concentration and undergoes a dynamic switch between acetylation and ubiquitylation. Under low glucose infiltration, HDAC1 maintains SRSF5 at a low acetylation level and recruits the ubiquitin ligase Smurf1 for ubiquitylation of SRSF5 on K125, resulting in the degradation of SRSF5 and reduced splicing towards CCAR1. The pro-apoptotic CCAR1L isoform brakes the cell proliferation and induces cell cycle arrest and even apoptosis (Fig. 10h, left). Under high glucose intake, the acetyl-transferase Tip60 specifically catalyzes the acetylation of SRSF5 on K125 and stabilizes SRSF5 proteins. Accumulation of SRSF5 increased splicing towards CCAR1 and more anti-apoptotic CCAR1S isoforms are produced to promote cell growth (Fig. 10h, right). This mutually exclusive acetylation and ubiquitylation pattern provides a precise and efficient on–off switch to control the cell fate to respond different energy environments. To our knowledge, the current findings establish the first linkage between RNA splicing machinery and glucose metabolism pathway.

Meeting the requirement for rapid proliferation of cancer cells, metabolic reprogramming provides more building blocks through glycolysis and gives the cancer cells an appropriate microenvironment by producing more lactate^32^. Of all the regulating mechanisms, protein acetylation could target and regulate almost all the metabolic enzymes^20,33,34^ and may serve as a wide bridge between the extracellular nutrient status and intracellular metabolic pathways. For example, PKM2 (pyruvate kinase M2 isoform) is acetylated by high glucose stimulation. This acetylation decreases PKM2 enzyme activity and promotes its lysosomal degradation, which results in the accumulation of glycolytic intermediate metabolites upstream of PKM2. This switch from ATP production to building block preparation promotes cell proliferation and tumor growth^35^. Furthermore, lactate dehydrogenase A (LDHA) acetylation decreases its enzymatic activity and promotes its degradation. In human pancreatic cancers, decreased levels of LDHA acetylation result in activation of LDHA and inhibition of LDHA degradation, eventually promotes cancer cell growth and migration^36^. These findings suggest that acetylation plays either positive or negative role in tumor development, depending on the cellular and molecular context. Despite the quintessential roles in almost every aspect of cellular activities, the spliceosome has long been considered as a housekeeping machinery that does not require significant post-translational regulation. This concept has remarkably changed with recent studies showing that the spliceosome activity and abundance are dynamically regulated by different posttranslational mechanisms, which are represented by multiple cellular events^37,38^. Our present work has provided a first glimpse at the dual regulation of ubiquitylation and acetylation regulation of the spliceosome key regulators during metabolic stress. The increased acetylation of SRSF5 in human lung cancers is conducive to stabilization of SRSF5 and activation of AS of CCAR1, which further promotes the tumor growth. Therefore, glucose-mediated metabolic response and oncogenic formation capacity are coordinated by the SRSF5-K125 acetylation event. The current findings add new splicing-associated mechanisms to elucidate how tumor cells utilize glucose to promote their growth and migration.

Smurf1 was originally identified as an inhibitory regulator of bone formation^39^. Subsequently, multiple functions of this ubiquitin ligase have been discovered^40^ in cell growth and differentiation^41^, cell migration^42^, cell polarity^43,44^, and autophagy^45^. *Smurf1*-knockout mice have a significant phenotype in the skeletal system^46^ and considerable manifestations during development and neural outgrowth^47^. A most recent study found that Smurf1 is a key sensor to synergize glucose uptake and osteoblast differentiation by targeting Runx2 for degradation^48,49^. Here, we identify Smurf1 as a bona fide ubiquitin ligase for K48-linked poly-ubiquitylation of SRSF5, thereby favoring proteasome-mediated degradation and weakened splicing ability when glucose concentration declines. It seems possible that HDAC1 and Smurf1 coordinate to control SRSF5 degradation under low glucose since the binding domains of Smurf1 or HDAC1 towards SRSF5 were non-overlapping. Whether HDAC1 and Smurf1 bind to SRSF5 simultaneously or successively upon glucose deprivation remains unrevealed. Additionally, in multiple tissues/organs of *Smurf1*-knockout mice, the expression of SRSF5 protein was higher than that in WT mice, suggesting that the Smurf1–SRSF5 axis might play diverse roles in vivo. Unravel other substrates in distinct microenvironments will be of great help in illustrating the functions of Smurf1.

Accumulating evidence has demonstrated that the well-known five modes of AS contributed to the onset and ongoing of tumors through different mechanisms including tumor invasion and metastasis^50^, epithelial to mesenchymal evasion, cancerous splicing of molecular markers (e.g., CD44 for cancer stem cells^51^) or classical tumor suppressors (e.g., BRCA and p53 for key cancerous pathways^52^). We here propose SRSF5 as a potent oncogenic factor, at least in the context of lung cancer development. Several lines of evidences support this notion. First, depletion of SRSF5 significantly reduced cell proliferation in cultured cells and tumor formation in xenografts. These effects are specific for SRSF5 in lung cancer since depletion of other SRSF members had no obvious effects (such as SRSF3 and SRSF10) or could not be rescued by CCAR1S (such as SRSF1). Second, depletion of SRSF5 reduced whereas overexpression of SRSF5 promoted glucose consumption and lactate production, rendering increased citrate level and acetyl-CoA level. Third, SRSF5 acetylation as well as total protein levels were upregulated in human lung cancers and correlated with cancer progression. Fourth, SRSF5 expression was positively correlated with more oncogenic CCAR1S and less pro-apoptotic CCAR1L, both in cancerous cell lines and in human lung cancer tissues. Our current findings suggest that SRSF5 promotes tumor growth largely dependent on the splicing of CCAR1, at least in lung cancer cells.

In summary, we demonstrated that the splicing factor SRSF5 plays a pivotal role in responding the glucose elevation and decline. Acetylation and ubiquitylation orchestrate to control SRSF5 stability and activity. SRSF5 regulates tumorigenesis of lung cancer cells through AS of CCAR1 pre-mRNA, and aberrant alternative splicing is a major contributor to cancer development. Therefore, the SRSF5–CCAR1 axis could be a potential target in lung cancer therapies.

## Methods

### Plasmids

6× Myc-Smurf1 wild-type, 6× Myc-Smurf1-C699A, Flag–Smurf1 were described previously^53^. Constructs of other Nedd4 family members were kindly presented by Dr. W.I. Sundquist as discussed before^54^. Hemagglutinin (HA)-tagged ubiquitin mutants with lysine retention at appropriate sites were kind gifts from Dr. H.R. Wang^55^. HA-CDKN2A was described previously^56^. HA-P300, Flag-Tip60, pcDNA-3.1-PCAF plasmids were obtained from Dr. W. Zhu. HDAC1-6 plasmids were kindly provided by Dr. Q.Y. Lei. Full-length SRSF5, GSDMA, MAPK1, Cullin 4B, ARHGEF1, RBM10, and hnRNPK were cloned by PCR amplification from cDNAs. SRSF5 RRM, RRMH, RS truncates; Smurf1, HDAC1 deletion mutants were sub-cloned into pCMV-Myc, pFlag-CMV-2 vectors as indicated. SRSF5 K125R, K125Q, K143R mutants; HDAC1 H178Y mutants and Tip60 G380E mutants were generated using the Quick-Change XL Site-Directed Mutagenesis Kit (Stratagene) according to the manufacturer’s instructions; GST-SRSF5 WT and truncates were constructed by sub-cloning the corresponding SRSF5 from Myc-SRSF5 into pGEX-4T-2, pGEX-6P-1 vectors as indicated. His-tagged Smurf1, Tip60, and GCN5 plasmids were described previously^57^. SRSF5-shRNA-resistant pQC-XIH-Flag-WT, pQC-XIH-Flag-K125R and pQC-XIH-Flag-K125RQ constructs as well as CCAR1-shRNA-resistant pQC-XIH-Flag-CCAR1L and pQC-XIH-Flag-CCAR1S constructs were generated using the Quick-Change XL Site-Directed Mutagenesis Kit (Stratagene) according to the manufacturer’s instructions, with specific primer sequences listed below to generate the silent mutations: K125R: Sense: 5′-CTGGCAGGATCTCAGAGATTTCATGAGACA-3′, Antisense: 5′-GACCGTCCTAGAGTCTCTAAAGTACTCTGT-3′; K125Q: Sense: 5′-CTGGCAGGATC TCCAAGATTTCATG AGACA-3′, Antisense: 5′-GACCGTCCTAGAGGTTCTAAAGTACTCTGT-3′; CCAR1L/S resistant: Sense: 5′-TTCCAGCACCCCGCTAGGCTAGTTAAG-3′; Antisense: 5′-TCCATCTCGAAGTTCTTGTGGGTCCTC-3′.

### ShRNAs and viral packing

ShRNAs constructs were cloned into the pLKO.1-puro lentivirus vectors or pMKO.1-puro retro-virus vectors including shSRSF5, shCCAR1L, shCCAR1S, which all employed two effective sequences as follows: Sh-SRSF5 (1#: Sense, 5′-GGTTACACCACATCATGAA-3′; 2#: Sense, 5′-TGAAGGAACGGTGTATGAA-3′). Sh-CCAR1L (1#: Sense, 5′-GGAGAAGATCCCTGGCATT-3′; 2#: Sense, 5′-GGACAAGAGTGCGCAA-3′). Sh-CCAR1S (1#: Sense, 5′-CCACATGACTTCAAGTA CTAT-3′; 2#: Sense, 5′-CCACAACACTACTCACTCCTAT-3′). Sh-CCAR1L/S (1#: Sense, 5′- AGCCA TCACTCCTTGGAGCAT-3′; 2#: Sense, 5′-TTCCAACATCCTGCTAGACTT-3′). Non-target control: 5′-TTCTCCGAACGTGTCACGT-3′. For putting back experiment-based retroviral production, A549 cells were infected with pMKO-sh-SRSF5 and pMKO-sh-Con retrovirus. 48 h later, SRSF5 knockdown of the stable cell line was collected after single drug selection (3 µg/ml puromycin, 1 week). Then, pQC-XIH-WT, pQC-XIH-K125R, pQC-XIH-CCAR1s, and pQC-XIH-CCAR1l retroviruses were added into the A549 shSRSF5 stable cell line as previously described^58^. 36 h later, positive cells stably expressing Flag-tagged WT, K125R, (putting back); CCAR1L, CCAR1S (rescue); retrovirus were collected and verified by immunoblotting after double drug selection (hygromycin, 3 µg/ml, 2 weeks). All viruses were used to infect cells in the presence of polybrene (8 µg/ml).

### siRNAs

For RNAi experiments, small interfering RNA (siRNA) oligos of *Smurf1*, *HDAC1*, *and Tip60* were carried out using commercial synthetic siRNA oligonucleotides (Shanghai GenePharma), and each target gene employed two effective sequences as below: si*Smurf1*-A (5′-GGGCUCUUCCAGUAUUCUATT-3′), si*Smurf*1-B (5′-GCAUCGAAGUGUCCAGAGAAG-3′); si*HDAC1*-A (5′-AACAGAAGCGUCCUGGA UUAGUU-3′), Si*HDAC1*-B (5′-GAGGCCAUCUUUGAGAUCAUCA-3′); si*Tip60*-A (5′-CGAAAC GGAAGGUGGAGG U-3′), si*Tip60*-B (5′-GAAGAUCCAGUUCCCCAAG-3′); Non-targeting siRNAs (5′-UUCUCCGAACGUGUCACGU-3′). All siRNA transfections were performed with Lipofectamine2000 (Invitrogen), and the knockdown efficiency was verified by quantitative PCR (qPCR) or western blot.

### Antibodies

All antibodies for normal immunoblot and immunoprecipitation were used at an optimal dilution in 5% non-fat milk. All antibodies were purchased as follows: Anti-SRSF5 for IB (ab67175, 1:400 dilution), anti-Smurf1 for IB (ab117552, 1:500) were purchased from Abcam. Anti-MEKK2 (sc1088, N-19, 1:200), anti-Smurf1 for IHC (sc25510, H60, 1:80), anti-Tip60 (N17, 1:100), anti-GST (sc-374171, A-6, 1:1000), anti-His (sc8036, H3, 1:300), anti-actin (sc-1616, 1:1000), Normal IgG (sc-2025, 1: 200) were purchased from Santa Cruz. Anti-HDAC1 (10E2, 5356), anti-Ub (P4D1, 3936), anti-AMPKα1/2 (2535 S, 1:1000), anti-phospho-AMPKα1/2 (Thr172) (5832S, 1:500), anti-ACC (3662S, 1:500), anti-phospho-ACC (Ser79) (3661S, 1:500), anti-S6K (2708, 1:400), anti-phospho-S6K(T389) (9234, 1:400), anti-mTOR (2983, 1:500) and anti-acetylated lysine antibody (9441, 1:400) were purchased from Cell Signaling. Anti-Flag (F7425, 1:1000), anti-Tubulin (T5168, 1:1000), peroxidase-conjugated anti-mouse secondary antibody (A4416), and peroxidase-conjugated anti-rabbit secondary antibody (A4914) were purchased from Sigma. IgG-linked Anti-Myc (M047-3, 1:1000) and Anti-HA (M180-3, 1:1000) were purchased from MBL. Anti-SRSF5 (H00006430-B01P, 1:150 for IHC) was purchased from Novus bio, Anti-CCAR1 (A300-435A) was purchased from Bethylab. Anti-SRSF1 (12929-I-AP, 1:1000), anti-SRSF2 (20371-I-AP, 1:500), anti-SRSF6 (11772-I-AP, 1:500), anti-SRSF9 (17926-I-AP, 1:500), anti-SRSF10 (10131-I-AP, 1:300), and anti-GFP (50430-2-AP, 1:2000) were purchased from Proteintech. Anti-SRSF3 (BS2559, 1:300), anti-SRSF4 (BS2750, 1:500), anti-SRSF7 (BS3954, 1:500), anti-SRSF8 (BS5952, 1:500), anti-SRSF11 (BS5927, 1:500), anti-SRSF12 (BS-21108R, 1:300) were all purchased from Bioworld. Antibody that specifically recognizing acetylation at lysine residue K125 (Ac-K125) was obtained commercially by immunizing rabbits at Shanghai Genomics, Inc. and the immunoblot assay are performed as previously^35^.

### Immunoprecipitation and immunoblotting

For immunoblots, cells were washed with PBS after 36–48 h transfection and lysed directly into EBC lysis buffer (50 mM Tris pH 7.5, 120 mM NaCl, 0.5% NP-40) supplemented with protease inhibitor cocktail (Complete mini, Roche) and phosphatase inhibitors (phosphatase inhibitor cocktail set I and II, Calbiochem) and resolved by SDS–polyacrylamide gel electrophoresis with corresponding concentration and immunoblotted with indicated antibodies. For immunoprecipitation assays, cells were lysed with HEPES lysis buffer (20 mM HEPES, pH 7.2, 50 mM NaCl, 0.5% Triton X-100, 1 mM NaF, and 1 mM DTT) or TNE lysis buffer (50 mM Tris–HCl pH 7.5, 150 mM NaCl, 1 mM EDTA and 1% Nonidet P40), 1000 µg lysates were incubated with the indicated antibody (1–2 μg) for 3–4 h at 4 °C followed by overnight incubation with Protein A/G agarose (Santa Cruz). Immuno-precipitates were washed three times with HEPES buffer or TNE buffer before being resolved by SDS-PAGE and immunoblotted with indicated antibodies. Cells were pretreated with 10 μM MG132 for 10–12 h to block the proteasome pathway before harvesting for immunoprecipitation experiments. Unprocessed original scans of blots are shown in Supplementary Fig. 9.

### Cell culture, cell transfection, and cell treatment

A549, H358, HepG2, HeLa, SMMC-7721, MCF7, and HEK293T cells were purchased from ATCC, and authenticated by STR profiling and tested for mycoplasma contamination by GENEWIZ. All cell lines besides A549 were cultured with DMEM medium supplemented with 10% fetal bovine serum (FBS), while A549 cell line was cultured with F12K medium with the same supplementary gradients. The WT and *Smurf1*^−/−^ MEFs were isolated and cultured as previously described^59^. Cells were transfected with various plasmids using Lipofectamine 2000 (Invitrogen) reagent or TuboFect in vitro transfection reagent (Fermentas) according to the manufacturer’s protocol. HDAC1-specific inhibitor Parthenolide (10 μM) was used as indicated. TSA (0.5 µM) and NAM (5 µM) were added to the culture medium 18 h and 6 h before harvest, respectively. Glucose-free medium was prepared with DMEM base (GIBCO, #11966) and supplemented with glucose (Sigma, G7528) as indicated.

### Glucose consumption and lactate production

Cells were seeded in culture plates and cultured for 8 h. The culture medium was then changed and cells were incubated for an additional 18 h. Glucose consumption in the culture medium were measured using the Glucose (GO) Assay Kit (KA4088, abnova) whereas lactate levels in the culture medium were determined using a Lactate Assay Kit (ab65331, abcam).

### Acetyl-CoA and citrate measurement

Acetyl-CoA content was tested according to the manufacturer’s protocols (Sigma, #MAK039) using fluorescence assay method. Briefly, 1 × 10^7^ cells were frozen rapidly (liquid N_2_) and pulverized. Samples were deproteinized by PCA precipitation; 2 ml 1 N perchloric acid (PCA) (Sigma, #34288) was added to the sample while the sample was kept cold. The sample was then homogenized thoroughly and centrifuged at 13,000*g* for 10 min to remove insoluble material. This supernatant was neutralized with 3 M potassium bicarbonate solution (Sigma, #60339), added in aliquots of 1 ml/10 ml of supernatant during vortexing, until bubble evolution ceased (2–5 aliquots). The samples were then cooled on ice for 5 min, and the pH was verified to be in the range of 6–8 in 1 ml of sample. Samples were spun for 2 min to pellet potassium bicarbonate. Reactions consisted of 20 μl sample solution, 21 μl acetyl-CoA assay buffers, 2 μl acetyl-CoA substrate mix, 1 μl conversion enzyme, 5 μl acetyl-CoA enzyme mix, and 2 μl fluorescent probe and were incubated at RT for 10 min. Fluorescence intensity was measured (*λ*_ex_ = 535/ *λ*_em_ = 587 nm) in black, 96-well flat-bottom plates with clear bottoms. The volumes of the 0.02 mM acetyl-CoA standard solution used to generate the standard curve were 0, 10, 20, 30, 40, and 50 μl.

Citrate was tested according to the manufacturer’s protocols (Bio Vision, #K655–100) using colorimetric assay method. Briefly, 2 × 10^6^ cells should be rapidly homogenized with 100 µl of Citrate Assay Buffer. Centrifuge at 15,000*g* for 10 min to remove cell debris. Deproteinizing samples using a perchloric acid/KOH protocol (BioVision, Cat. #K808–200). Add 1–50 µl sample into duplicate wells of a 96-well plate and bring volume to 50 µl with Assay Buffer. After development and incubation, OD value was measured at 570 nm.

### In vitro acetylation assays

Distinct cDNA encoding truncated forms of SRSF5 protein were fused in frame with GST by sub-cloning into pGEX-6P-1 plasmid. Beads coated with GST, GST–SRSF5 (1–80), GST–SRSF2 (80–180), GST–SRSF2 (180–273) fusion proteins were prepared according to the manufacturer’s protocol (Bulk GST Purification module, Pharmacia Biotech). “In vitro” acetyltransferase assays were performed using 2 mg of each recombinant GST–SRSF5 fusion peptides, 1 mg of recombinant His-tagged Tip60^(212–513)^ or His-tagged GCN5 protein, and 0.05 mCi of [^14^C]acetyl-CoA, as described previously^60^.

### Tip60 HAT activity assays

Tip60 proteins were purified by immunoprecipitation with Tip60 antibody from A549 cells maintained in various glucose concentration. The Tip60 HAT activity was assayed by incubating 5 ml of [^3^H]-acetyl CoA (Perkin Elmer) and 0.5 mg of recombinant histone H4 (Biolabs) with or without purified Tip60 (5 mg) in a buffer containing 50 mM Tris–HCl (pH 8.0), 1 mM DTT, 0.1 mM EDTA, and 10% glycerol for 1 h at 30 °C. To measure Tip60 autoacetylation, reaction mixtures were resolved using SDS-PAGE, and the level of [^3^H]-acetyl-labeled Tip60 and the level of acetylated histones was determined by immunoblot using anti-acetyl-lysine antibody.

### HDAC1 deacetylase activity assays

Non-radiolabeled acetylated histones were prepared from cultured cells treated with 5 mM sodium butyrate overnight. The deacetylase activity for acetylated HDAC1 or core histones were assayed by incubating 50 ng HDAC1 with 200 ng acetylated histones at 30 °C for 30 min in 15 µl of HDAC buffer (25 mM Tris, pH 8.0, 137 mM NaCl, 2.7 mM KCl, 1 mM MgCl_2_). The reaction was stopped by addition of 50 µl stop buffer (1.44 M HCl, 0.24 M HOAc) and the products were then subjected to western blot analysis with anti-acetyl-lysine antibody.

### GST pull-down assays

Full-length SRSF5 as well as truncates were inserted into the pGEX-6P-1 vector (GE healthcare). Smurf1 was inserted into the pET-28a vector (Novagen). To detect the direct binding, bacteria-expressed GST-tagged proteins were immobilized on glutathione-Sepharose 4B beads (GE Healthcare) and then incubated with His-tagged proteins for 8 h at 4 °C under rotation. Beads were washed with GST-binding buffer (100 mM NaCl, 50 mM NaF, 2 mM EDTA, 1% NP-40, and protease inhibitor mixture) and proteins were eluted, followed by immunoblotting.

### RT-PCR and quantitative PCR

Total cell RNA was prepared using Trizol reagent (Invitrogen) following manufacturer’s instructions. Briefly, total RNAs were extracted from indicated cell lines and reverse transcription was performed using oligo (dT) priming and M-MLV Reverse Transcriptase according to the manufacturer’s instructions (Promega). Primers used for the indicated gene products are listed in Supplementary Table 3. Quantification of all gene transcripts was carried out by real-time PCR using SYBR Premix Ex Taq kit (TaKaRa, Otsu, Shiga, Japan), β-actin was used as internal control.

### Protein degradation analysis

For SRSF5 half-life assay, Lipofectamine-2000 transfection was performed when A549 cells or HEK293T cells in 2 cm plates reached about 60% confluence. Plasmids encoding SRSF5 WT and mutants (K125R, K125Q); Smurf1, HDAC1, Tip60, or their corresponding siRNAs were used in transfection as indicated in individual experiments. 24 h later, cells were treated with the protein synthesis inhibitor cycloheximide (Sigma, 10 μg ml^−1^) for the indicated durations before harvest.

### In vivo ubiquitylation assays

For SRSF5 ubiquitylation analysis, HEK293T cells were transfected with HA-ubiquitin, Myc-SRSF5, Flag-Smurf1 as indicated. At 36 h after transfection, cells were lysed in Tris-HCl buffer (0.1% SDS 0.5 mM EDTA, 1 mM dithiothreitol [DTT], 150 mM Tris [pH 7.5]) and then incubated with anti-Flag or anti-Myc antibody for 3 h and protein A/G-agarose beads for a further 8 h at 4 °C. After three washes, ubiquitinated SRSF5 was detected by immunoblotting with anti-HA monoclonal antibody. For detection of endogenous ubiquitylation of SRSF5, *Smurf1*^+/+^, and *Smurf1*^−/−^ MEF cells were treated with the proteasome inhibitor MG132 (20 µM; Sigma) for 10 h, and proteins were immunoprecipitated with the appropriate antibody (SRSF5, Abcam, 2 µg) followed by immunoblotting with anti-ubiquitin antibody (CST).

### In vitro ubiquitylation assays

His-Smurf1s, Wild-type, CA (C699A), GST, GST–SRSF5 were expressed in *Escherichia coli* BL21 and purified. Indicated proteins were pretreated at 30 °C for 30 min. Afterwards, 0.7 µg of E1, 0.9 µg of Ubc-H5c, 12 µg of HA-ubiquitin, 0.7 µg of His-Smurf1, and 1.6 µg of GST or GST-SRSF5 of ubiquitylation assay buffer. The reactions were stopped by the addition of SDS-PAGE sample buffer. The reaction products were resolved by SDS-PAGE gel and probed with the indicated antibodies.

### Apoptosis assays

For detection of apoptosis, cells with different treatments were co-stained with Annexin-V-PE and 7-AAD (Annexin V-PE, Apoptosis Detection Kit I, BD Bioscience) according to the manufacturer’s instructions. Samples were analyzed on a FACS Calibur (BD Biosciences). Data were analyzed with the FlowJo software (Treestar).

### Cell proliferation and colony-survival assay

Cell proliferation was measured by counting the number of the cells. Briefly, triplicate plates of cells were trypsinized and stained with Trypan blue, and unstained cells were counted using a haemo-cytometer. Colony-survival assay was performed as previously described^61^. Briefly, cells were seeded sparsely (2000 cells per well for 6-well plates, 1000 cells per well for 12-well plates) and were grown for 10–15 days with normal medium. Colonies were fixed and stained with 0.5% crystal violet solution in 20% methanol. Pictures of the plates were taken using Canon G9 digital camera and the number of colonies formed under each condition was scored from six random areas for each group.

### Mouse xenograft assays

BALB/c male nude mice (4–5 weeks old at purchase, 18.0 ± 2.0 g) were randomly divided into indicated groups and maintained in pathogen-free conditions. 3 × 10^6^ cells were mixed with Matrigel (0.25 v/v) and injected subcutaneously with indicated cells at each flank. Tumor size was measured twice a week with a caliper, and the tumor volume was determined with the formula: *L* × *W*^2^ × 0.52, where *L* is the longest diameter and *W* is the shortest diameter. After 28 days, mice were euthanized and in vivo solid tumors were dissected and tumor weights were measured and recorded. Hematoxylin and eosin (H&E) staining were reviewed to ensure the cancer tissue and normal tissue.

### Immuno-histochemical staining

Xenografts harvested from three mice of each treatment group were formalin-fixed and paraffin-embedded as previously^62^. Excised tumors from indicated groups were fixed in 4% buffered formalin for 16 h and processed by the conventional paraffin-embedded method. To determine the apoptotic cells in tumors, the paraffin-embedded tumor sections (3.5 µm thick) were stained with the TUNEL kit (Promega Corporation) following the manufacturer’s instruction. The nucleus of the cells was stained with propidium iodide. We performed staining of SRSF5-Total, Smurf1, SRSF5-Ac-K125, on the same paraffin-embedded tissue blocks that were used for clinical diagnosis. Immunohistochemistry was performed using the avidin–biotin complex method (Vector Laboratories), including heat-induced antigen-retrieval procedures. Briefly, antigen retrieval was done by incubating the sections in 10 mM citrate buffer (pH 6.0) at high wave in a microwave for 10 min followed by the incubation with 5% block serum for 0.5 h. Incubation with polyclonal antibodies against Smurf1 (H60, 1:50 dilution, Santa Cruz), SRSF5 (H00006430-B01P, 1:100 dilution, Novus Bio), Ac-K125 (Shanghai Genomics; 1:50 dilution) was performed at 4 °C for 14 h. Quality assessment was performed on each batch of slides by including a negative control in which the primary antibody was replaced by 10% normal goat serum to preclude non-specific signals. Staining was assessed by pathologists who were blinded to the sample origins and the patient outcomes. Image acquisition and processing software were performed using an Olympus DP12 camera and software, and Adobe Photoshop 6.0 and representative field were photographed under 400× (20 µm) magnifications.

### Human lung cancer samples

Sixty paired primary lung cancer and their corresponding adjacent normal tissues were obtained from lung cancer patients treated at General Hospital of PLA were obtained. Sample collection was approved by the Research Ethics Committee at the General Hospital of PLA and written informed consent was obtained from all subjects or their relatives. The fresh samples were snap-frozen in liquid nitrogen and stored at −80 °C until further analysis.

### mRNA-sequencing and gene expression analysis

For mRNA-seq displayed in Supplementary Fig. 2, experiments were performed by Novogene (Beijing). mRNA-seq library is prepared for sequencing using standard Illumina protocols. Total RNA samples from A549 cells with or without *SRSF5* knockdown in three biological repeats were isolated using TRIzol reagent (Invitrogen) and treated with RNase-free DNase I (New England Biolabs, MA, USA), to remove any contaminating genomic DNA. mRNA extraction is performed using Dynabeads oligo(dT) (Invitrogen Dynal). Double-stranded complementary DNAs are synthesized using Superscript II reverse transcriptase (Invitrogen) and random hexamer primers. The cDNAs are then fragmented by nebulization and the standard Illumina protocol is followed thereafter, to create the mRNA-seq library. For the data analysis, basecalls are performed using CASAVA. Reads are aligned to the genome using the split read aligner TopHat (v2.0.7) and Bowtie2, using default parameters. HTSeq is used for estimating their abundances. The original sequence data have been submitted to the database of the NCBI Sequence Read Archive (http://trace.ncbi.nlm.nih.gov/traces/sra) under the accession number SRP119739.

For mRNA-seq revealed in Fig. 3, total RNAs from Sh-Ctrl, Sh-CCAR1L, and Sh-CCAR1S A549 cells with three biological repeats were isolated using TRIzol reagent (Invitrogen) and treated with RNase-free DNase I (New England Biolabs, MA, USA). Library construction and sequencing were performed on a BGISEQ-500 by Beijing Genomic Institute (www.genomics.org.cn, BGI, Shenzhen, China). Clean-tags were mapped to the reference genome and genes available with a perfect match or one mismatch. The original sequence data have been submitted to the database of the NCBI Sequence Read Archive (http://trace.ncbi.nlm.nih.gov/Traces/sra) under the accession number SRP119820.

For gene expression analysis, the matched reads were calculated and then normalized to RPKM using RESM software. The significance of the differential expression of genes was defined by the bioinformatics service of BGI according to the combination of the absolute value of log2-ratio ≥ 1 and FDR ≤ 0.001. KOG functional classification, Gene Ontology (GO), and pathway annotation and enrichment analyses were based on the NCBI COG (https://www.ncbi.nlm.nih.gov/COG/), Gene Ontology Database (http://www.geneontology.org/), and KEGG pathway database (http://www.genome.jp/kegg/), respectively. The software Cluster and Java Treeview were used for hierarchical cluster analysis of gene expression patterns.

### LC-MS/MS analysis

Distinct isoforms of CCAR1 were immunoprecipitated with Protein A-G agarose from A549 cells stably expressing Flag-CCAR1L and Flag-CCAR1S with endogenous CCAR1L/S depleted. Proteins in IP samples were precipitated with IP buffer (50 mM Tris–HCl pH 8.0, 100 mM NaCl, 1 mM EDTA and 1% Nonidet P40) and then washed three times with TNE buffer. The samples from in-gel digestion were analyzed on a Q-Exactive HF MS (Thermo Fisher Scientific) interfaced with an Easy-nLC 1,200 nanoflow LC system (Thermo Fisher Scientific). Tryptic peptides were dissolved with 10 μl of loading buffer (5% methanol and 0.1% formic acid), and 5 μl was loaded onto a homemade trap column (2 cm) packed with C18 reverse-phase resin (particle size, 3 μm; pore size, 120 Å; SunChrom, USA) at a maximum pressure of 280 bar with 12 μl of solvent A (0.1% formic acid in water). Peptides were separated on a 150 μm × 12 cm silica microcolumn (1.9 μm C18, homemade) with a linear gradient of 5–35% Mobile Phase B (80% acetonitrile and 0.1% formic acid) at a flow rate of 600 nl min^−1^ for 75 min. The MS analysis was performed in a data-dependent manner with full scans (*m*/*z* 300–1400) acquired using an Orbitrap mass analyzer at a mass resolution of 120,000. Up to 20 of the most intense precursor ions from a survey scan were selected for MS/MS and detected by the Orbitrap at a mass resolution of 15,000. All the tandem mass spectra were acquired using the higher-energy collision dissociation (HCD) method with normalized collision energy of 27%. The parameter settings were: automatic gain control for full MS was 3e6, and that for MS/MS was 5e4, with maximum ion injection times of 80 ms and 20 ms, respectively. Dynamic exclusion time was 12 s, and the window for isolating the precursors was 1.6 *m*/*z*.

For further MS analysis, raw files were searched against the human refseq protein database (32,014 proteins, version 04/07/2013) with Proteome Discoverer (Thermo Fisher Scientific, version 1.4) using the MASCOT search engine with percolator. The mass tolerance of the precursor ions was set to 20 p.p.m. For the tolerance of the product ions, QE HF was set to 50 mmu. Up to two missed cleavages were allowed for protease digestion, and the minimal required peptide length was set to seven amino acids. N-terminal protein acetylation and methionine oxidation were set as variable modifications. The data were also searched against a decoy database so that protein identifications were accepted at a FDR of 1%. Protein identification data (accession numbers, peptides observed, sequence coverage) are available in Supplemental Data 1, respectively. All raw data and search results have been deposited to the PRIDE database (http://www.iprox.org/index) with the accession number: PXD009266. Please access the raw files with user ID “reviewer60237@ebi.ac.uk” and password “OveWSRwF”.

### Statistical analysis

All results are shown as the mean ± s.e.m. of multiple independent experiments. Detailed *n* values for each panel in the figures are stated in the corresponding legends. A student’s *t*-test, a Mann–Whitney test (for two group comparisons) or a Kruskal–Wallis one-way ANOVA followed by Dunn’s multiple comparison tests (for more than two group comparisons) were used for statistical analyses. All statistical analyses were performed with GraphPad Prism 5 and SPSS 19.0 software. All statistical tests were two-sided, and *P* values < 0.05 were considered to be statistically significant.

In the nude mouse experiments, the mice were randomly assigned and the investigators were blinded to experiments and outcome assessment during nude mouse experiments. Other experiments were not randomized for the investigators were not blinded to allocation during experiments and outcome assessment.

### Ethics statement

All animals were handled in strict accordance to the "Guide for the Care and Use of Laboratory Animals" and the "Principles for the Utilization and Care of Vertebrate Animals", and all animal work was approved by the Institutional Animal Care and Use Committee (IACUC) at the Beijing Institute of Radiation Medicine.

### Data availability

The original RNA-seq data of SRSF5 knockdown in A549 cells have been deposited in the database of the NCBI Sequence Read Archive (http://trace.ncbi.nlm.nih.gov/Traces/sra) under the accession number SRP119739. The original RNA-seq data of CCAR1S and CCAR1L knockdown in A549 cells have been deposited in the database of the NCBI Sequence Read Archive (http://trace.ncbi.nlm.nih.gov/Traces/sra) under the accession number SRP119820. For IP-MS of CCAR1L and CCAR1S, all raw data and search results have been deposited to the PRIDE database (http://www.iprox.org/index) with the accession number: PXD009266. Please access the raw files with user ID “reviewer60237@ebi.ac.uk” and password “OveWSRwF”. The authors declare that all the relevant data supporting the findings of this study are available within the article and its Supplementary Information files, or from the corresponding author on reasonable request.

## Electronic supplementary material

Supplementary Information

Description of Additional Supplementary Files

Supplementary Data 1

Supplementary Data 2
