# Supplementary material for: Mutually exclusive acetylation and ubiquitylation of the splicing factor SRSF5 control tumor growth
Source: Nat Commun. 2018 Jun 25;9:2464. doi: 10.1038/s41467-018-04815-3 (PMC6018636; doi:10.1038/s41467-018-04815-3)
Supplement: Supplementary file 1 — Supplementary Information [file 41467_2018_4815_MOESM1_ESM.pdf]

# Mutually Exclusive Acetylation and Ubiquitylation of the Splicing Factor SRSF5 Control Tumor Growth

Yuhan Chen et al.

# Supplementary Figure 1. SRSF5 is stabilized at high glucose to promote tumorigenesis

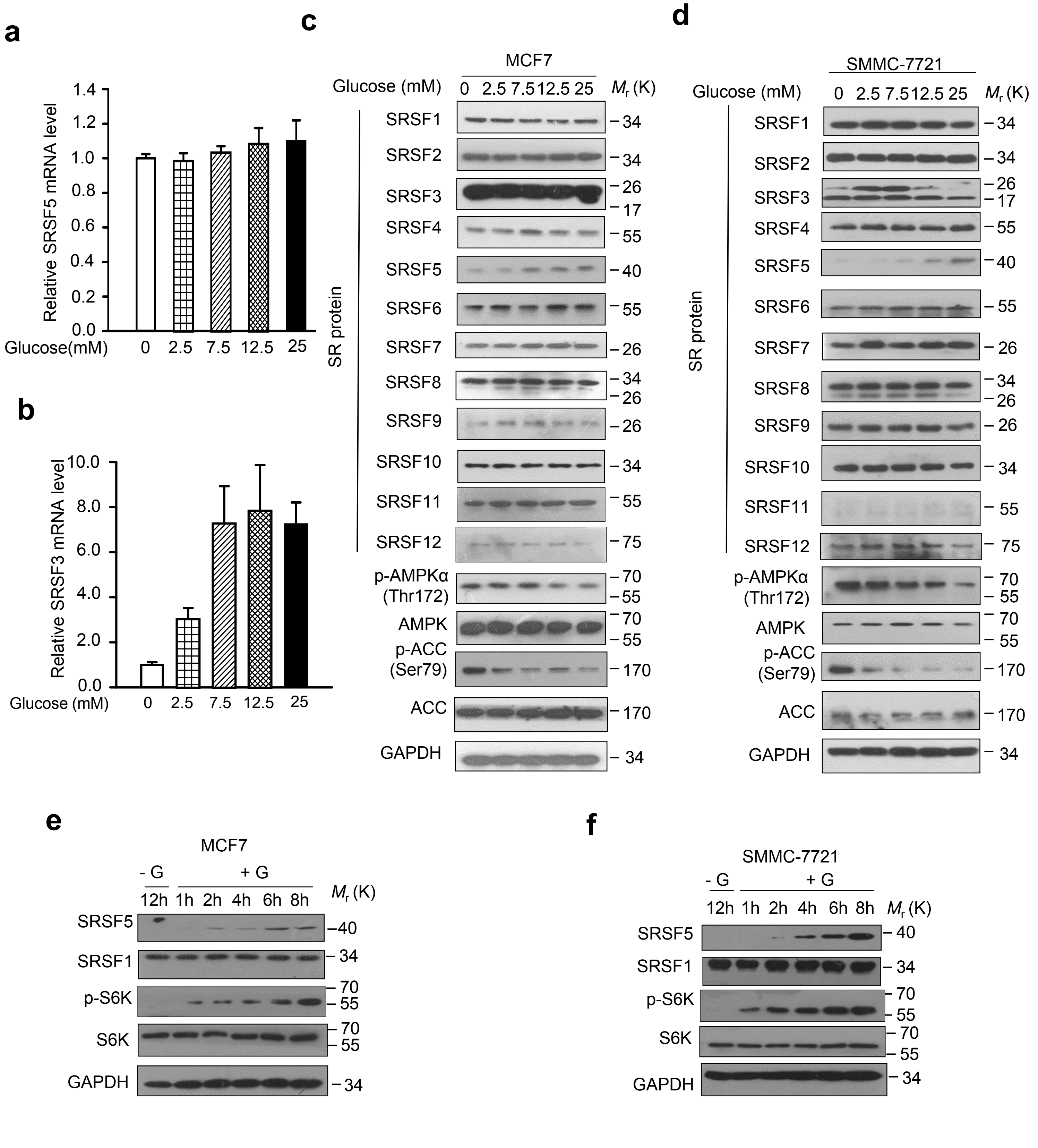


**Supplementary Figure 1. SRSF5 is stabilized at high glucose to promote tumorigenesis. a**, **b**, A549 cells were maintained at indicated concentrations of glucose. Quantitative PCR was performed to determine the relative mRNA level of SRSF5 **(a)** or SRSF3 **(b)**. Analysis of each gene was normalized to the gene *Gapdh* (unpaired two-tailed Student's *t* test). **c, d,** Additional cell lines were administrated to glucose titration as in **(a)** to determine the generic regulatory effect of SRSF5. MCF7 **(c)** and SMMC-7721 **(d)** cells were maintained at indicated concentrations of glucoses and the abundance of indicated SRSFs were determined by immunoblotting. **e**, **f,** The expression of SRSF5 was regulated by glucose deprivation and stimulation. Multiple cancer cell lines including MCF7 **(e)** and SMMC-7721 **(f)** were glucose starved for 12 h and then stimulated with glucose (25 mM) for the indicated times. Cell lysates from each time point were subjected to immunoblotting. Data are representative of three independent biological replicates (**a** and **b**; mean and s.e.m., n = 3).

# Supplementary Figure 2. SRSF5 controls CCAR1 splicing to regulate tumor cell growth

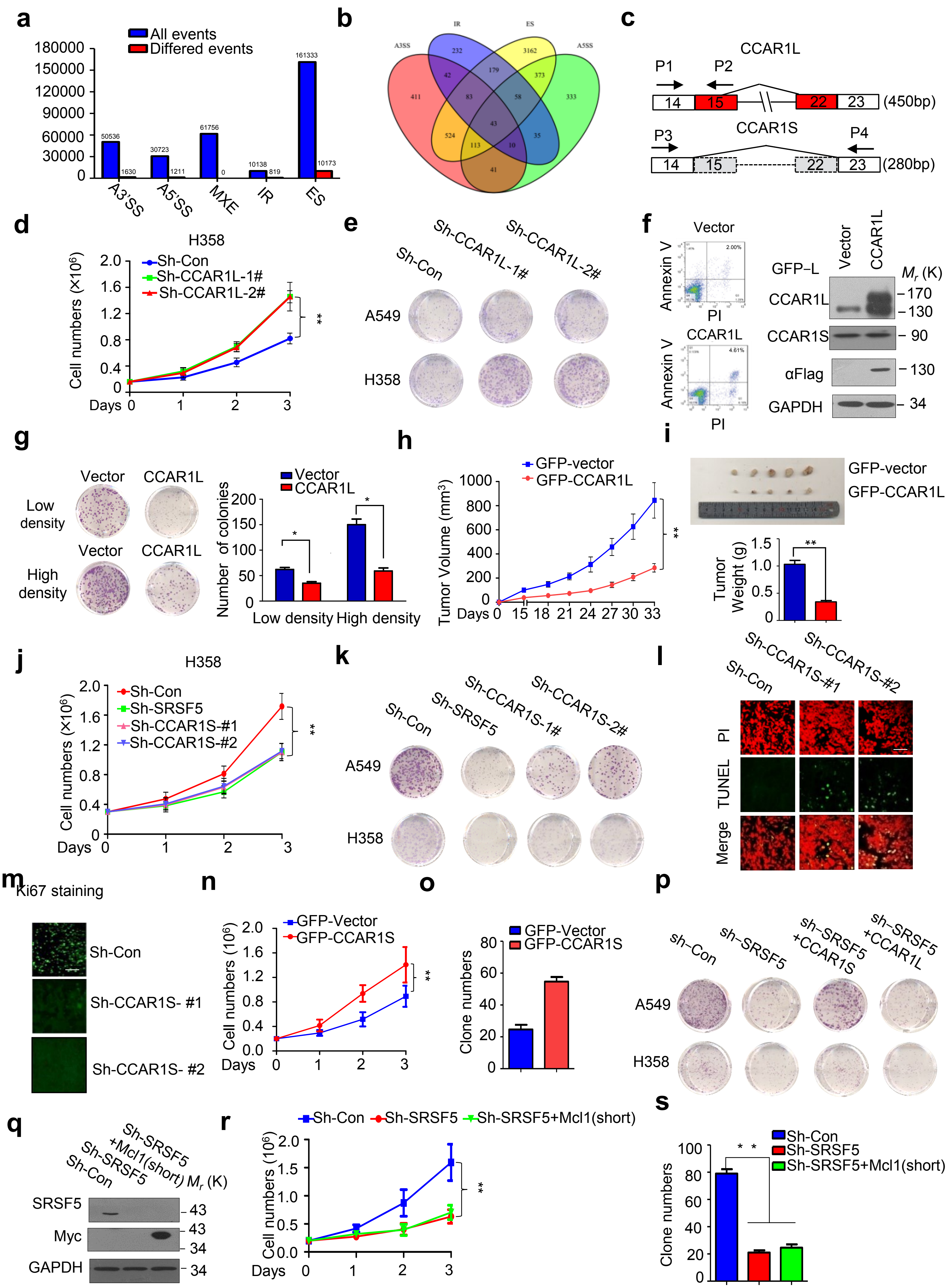

**Supplementary Figure 2. SRSF5 controls CCAR1 splicing to regulate tumor cell growth.** **a**, Differentially alternative splicing patterns in SRSF5 knockdown vs wild-type cells. **b**, Overlap of four types of AS genes illustrated in five-way Venn diagrams of SRSF5 knockdown vs wild-type cells. Significant splicing differences were identified following the criteria of an absolute value of percent spliced in cutoff ≥ 0.20 plus false discovery rate (FDR) ≤ 0.001. **c**, Diagrams for detection of CCAR1 variants. **d**, **e**, Expression of CCAR1L was knockdown in H358 cells with two independent shRNAs. Cell proliferation assay **(d)** and standard clonogenic survival assay **(e)** were performed (*$P$ < 0.05, two way ANOVA-test). **f**, A549 cells were stably transfected with GFP-CCAR1L encompassing virus and overexpression of CCAR1L was confirmed by immunoblotting (left panel) Stable overexpression of CCAR1L increases apoptosis rate in A549 cells (right panel). Clonogenic survival assay **(g)** and xenograft tumor model assay were conducted with the above described cells. **h**, **i**, After cell inoculation, tumors were measured **(h)** followed by excision after six weeks and weighted **(i)**. The two-tailed Student's t-test was used. *$P$ < 0.05; **$P$ < 0.01; ***$P$ < 0.001**. j-m**, H358 cells were infected with sh-Con and sh-CCAR1S lentivirus particles and Cell proliferation assay were performed as in Fig. 2g **(j)**. Clonogenic survival assay **(k)**, TUNEL assay **(l)** and Ki67 staining **(m)** were conducted. **n**, **o**, Cell proliferation assay **(n)** and clonogenic formation assay **(o)** of A549 cells either stably expressing pCDH-vector or pCDH-CCAR1S were performed (**$P$ < 0.01, two-way ANOVA test). **p**, the representative images of Clonogenic survival assay of A549 and H358 cells as described in Fig. 2l are shown. q. Identification of SRSF5 knocking down and SRSF5-depleted plus Myc-Mcl-1S overexpressing cells. **r**, **s**, Cell proliferation assay **(r)** and clonogenic survival assay **(s)** were performed with the above described cells (**$P$ < 0.01, two way ANOVA-test). Data are representative of three independent biological replicates (**d, e, g, j, k, n, o, p, r, s**; mean and s.e.m., n = 3). Scale bar, 50 μm.

# Supplementary Figure 3. Network analysis of potential CCAR1L/S-associated proteins

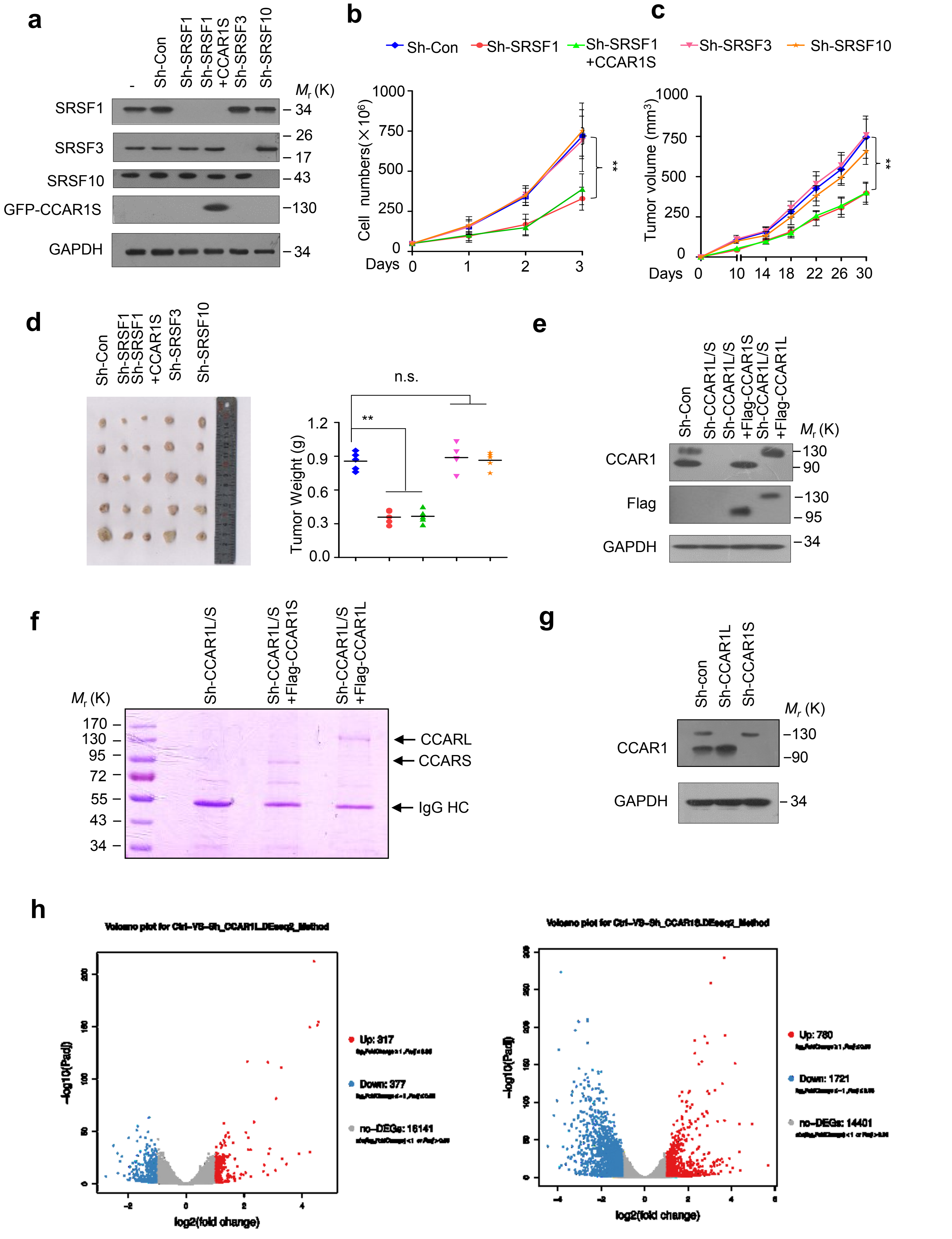

# Supplementary Figure 3 (continuation)

**i**

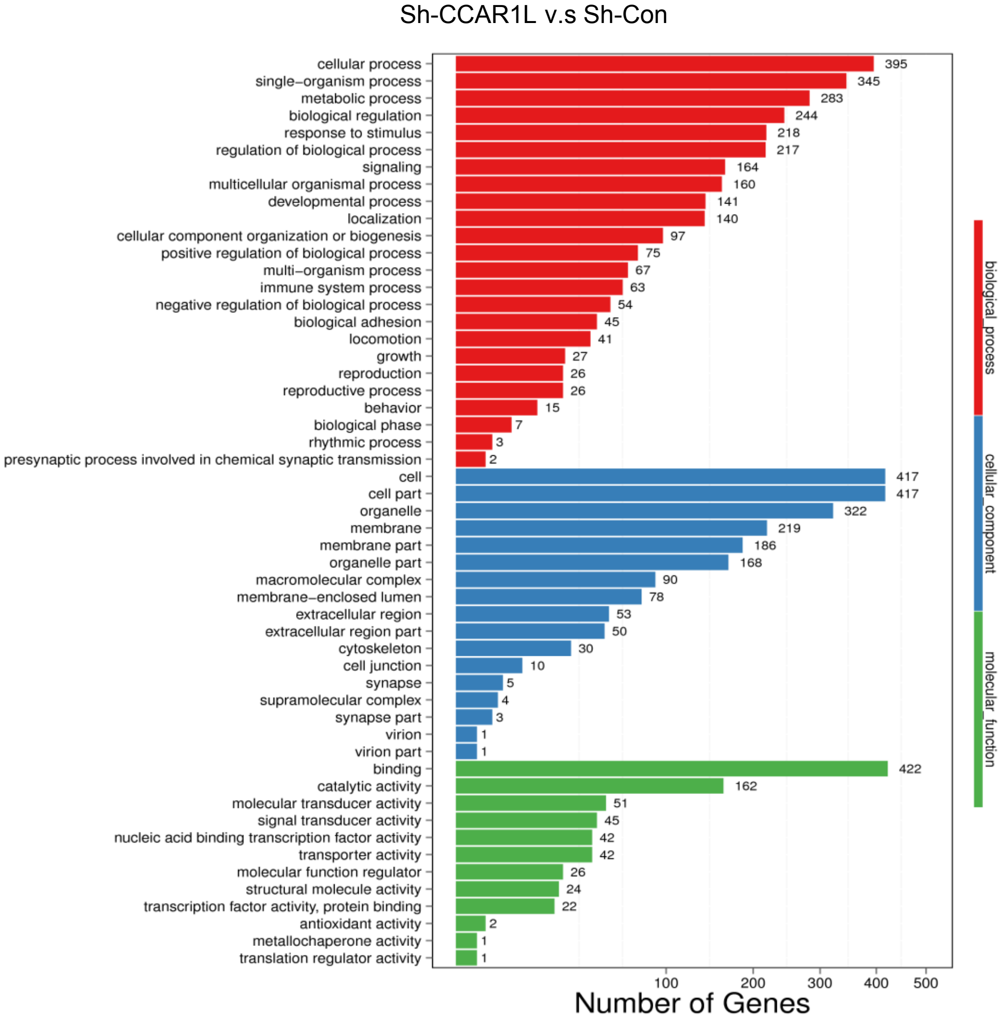


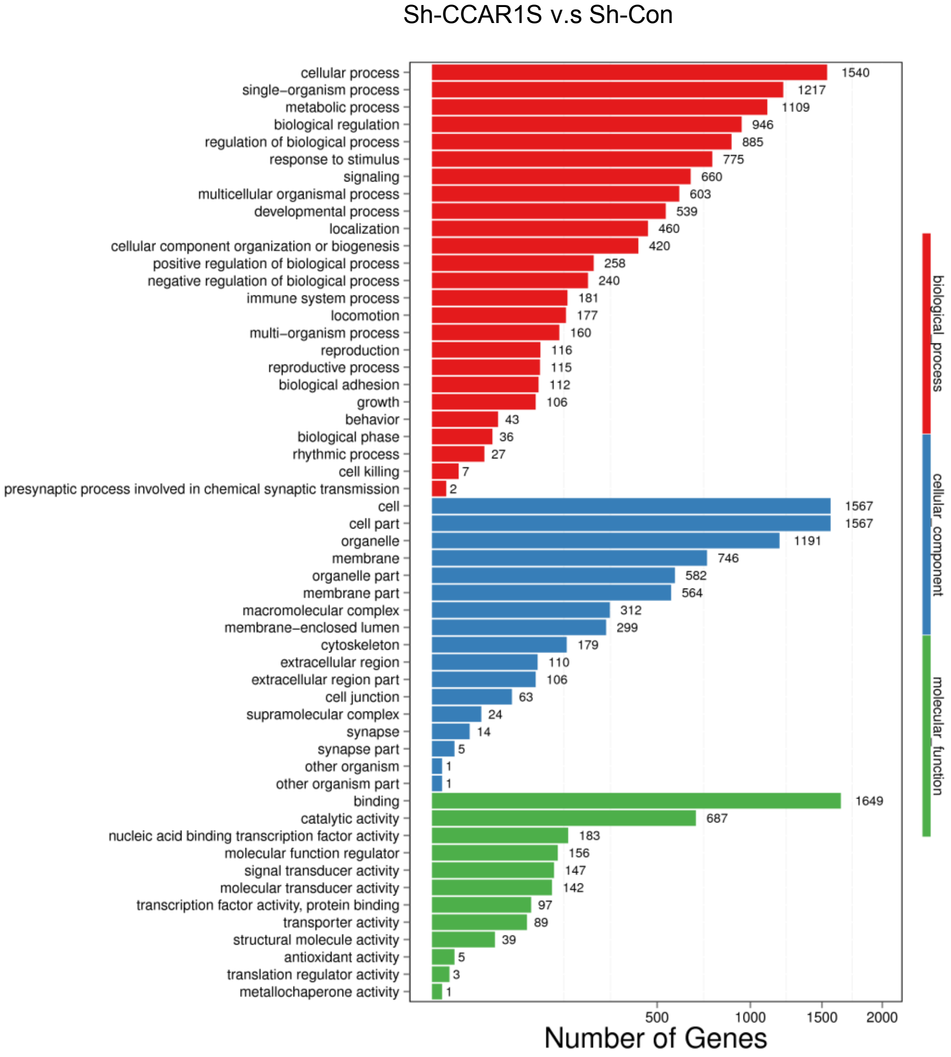

**Supplementary Figure 3. Network analysis of potential CCAR1L/S-associated proteins**
**a**, Identification of SRSF1, SRSF3 or SRSF10 knocking down and SRSF1-depleted plus CCAR1S overexpressing cells. Whole cell extracts were prepared from indicated cell pools. The knockdown efficiency and re-expression were determined by immunoblotting analysis. **b**, Cell proliferation assay were performed with the above described cells (**$P < 0.01$, two way ANOVA-test). **c**, **d**, Subcutaneous xenograft experiment was performed in nude mice using the indicated A549 stable cells. Major and minor diameters of tumors were measured and tumor volumes were calculated. The two-tailed Student's t-test was used. **$P < 0.01$; (c). Thirty days after injection, tumors were dissected, photographed and weighted. The two-tailed Student's t-test was used. **$P < 0.01$ (d). **e**, Verification of A549 stable cell lines. Knockdown efficiency and re-expression levels of CCAR1L or CCAR1S were determined by immunoblotting. **f**, Commassie blue staining validating the immunoprecipitation of stably over-expressed Flag-tagged CCAR1L and Flag-tagged CCAR1S. **g**, Identification of CCAR1L and CCAR1S specifically depleted cells. Whole cell extracts of A549 cells were prepared from indicated cell pools. The knockdown efficiency was determined by immunoblotting analysis. **h**, Identification of the differentially expressed proteins were graphed in volcano Plot. The log2 mean ratio of three replicates was plotted against the corresponding -log10 p value. Proteins that exhibited fold change greater than two folds and P value ≤ 0.05 were considered up-regulated and marked with red. Proteins that decreased more than two folds and had P values ≤ 0.05 were considered downregulated and marked with blue. The gray proteins were not significantly changed. **i**, Gene Ontology (GO) enrichment analysis for biological process, cell component and molecular function of up-regulated proteins. The -log10 p value of enrichment is shown on x axis; the number of associated proteins for each term is shown on y axis. Data are representative of three independent biological replicates (**b**; mean and s.e.m., n = 3).

# Supplementary Figure 4. SRSF5 regulates glucose metabolism and acetyl-CoA production

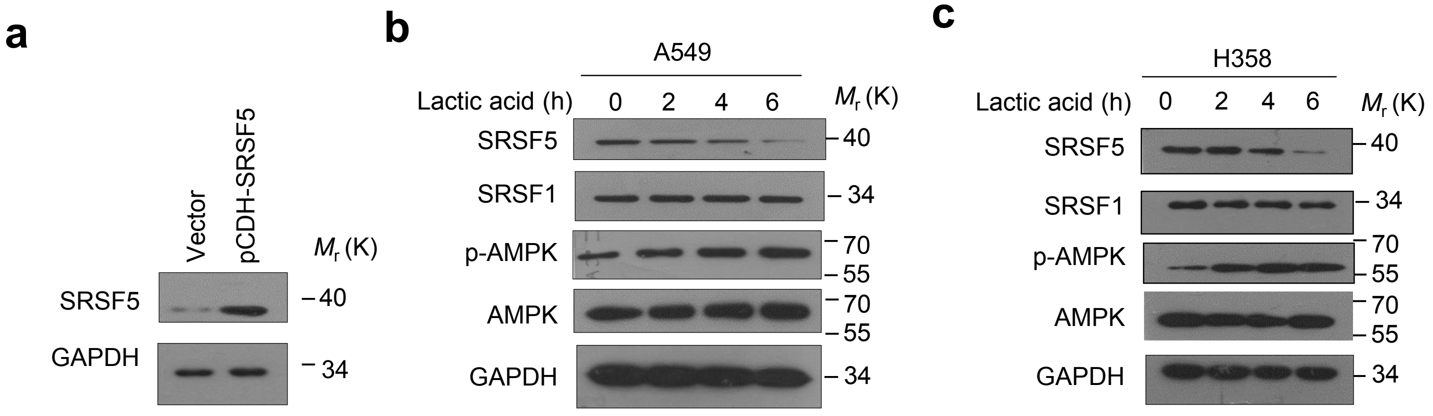


**Supplementary Figure 4. SRSF5 regulates glucose metabolism and acetyl-CoA production**
**a**, Stable over-expression of SRSF5 in A549 cells were confirmed by immunoblotting. **b**, **c**, Lactic acidosis triggers the inhibition of SRSF5 expression in A549 **(b)** and H358 **(c)** cells and the activation of AMPK (phosphorylation at Thr 172) were determined by immunoblotting.

# Supplementary Figure 5. Tip60 acetylates SRSF5 under high glucose

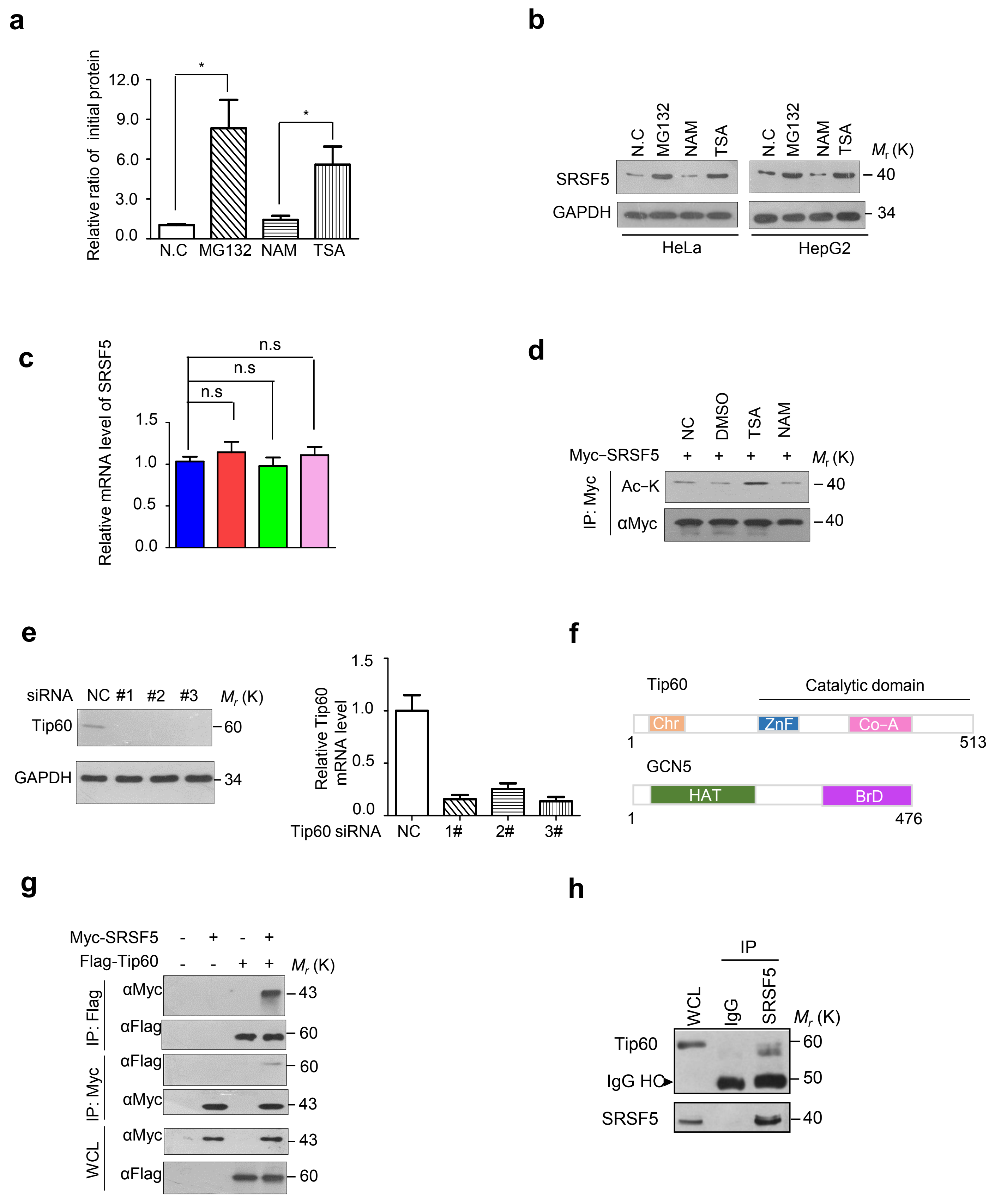

**Supplementary Figure 5. Tip60 acetylates SRSF5 under high glucose**

**a**, Quantification of the SRSF5 protein levels relative to GAPDH for Fig. 5a (*: $P < 0.05$, Student's *t* -test). **b**, HeLa and HepG2 cells were treated with different deacetylase inhibitors as described in Fig. 5a. Endogenous protein level of SRSF5 was detected by immunoblotting analysis. **c**. The mRNA expression level of SRSF5 remains unchanged after stimulation of MG132, TSA or NAM. Quantitative-PCR analysis of SRSF5 mRNA level was performed upon different treatment. (n.s.: no significance, two-way ANOVA test). **d**, Exogenous SRSF5 is acetylated. Same amount of Myc-SRSF5 plasmids were transfected into HEK293T cell lines followed by indicated treatments. The acetylation level of Myc-SRSF5 were determined by immunoblotting analysis. **e**, *Tip60* knockdown efficiency was measured by quantitative PCR and immunoblotting analysis. (*$P < 0.05$, student's t-test) **f**, Diagrams depicting the catalytic domain of Tip60 for *in vitro* acetylation assay whereas GCN5 was used a negative control. **g**, Exogenous interaction between SRSF5 and Tip60. Immunoblotting analysis of WCL and anti-Myc or anti-Flag immunoprecipitates derived from HEK293T cells transfected with Myc-SRSF5 and Flag-Tip60.**h**. Endogenous interaction between SRSF5 and Tip60 were determined by co-immunoprecipitation assays and immunoblotting analysis. Data are representative of three independent biological replicates (**a**, **c**, **e**; mean and s.e.m., n = 3).

# Supplementary Figure 6. Smurf1 targets SRSF5 for degradation upon low glucose intake

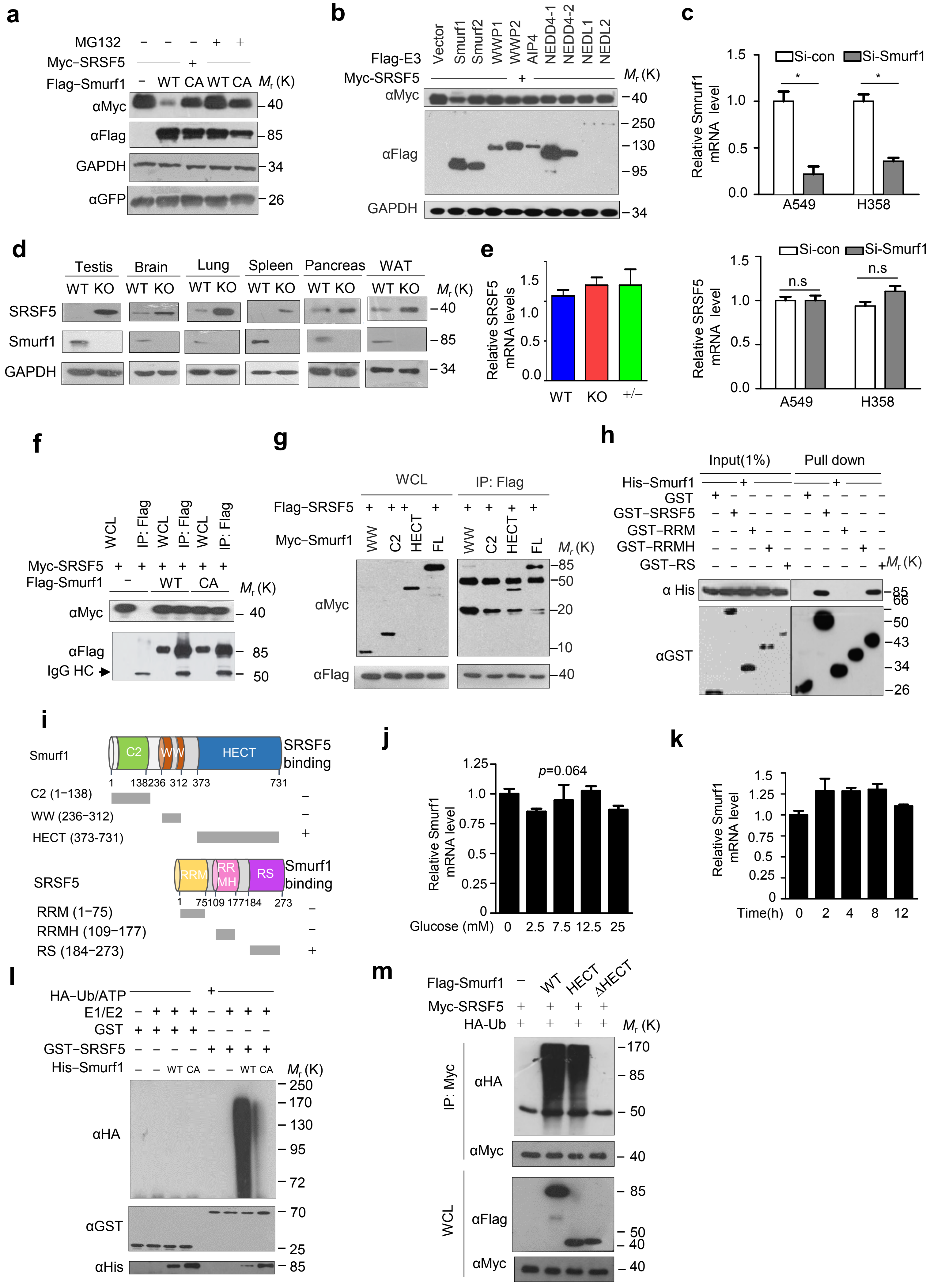

**Supplementary Figure 6. Smurf1 targets SRSF5 for degradation upon low glucose intake a**, E3 ligase activity of Smurf1 was indispensable for its negative regulatory effect upon SRSF5. **b**, Flag-tagged E3 ligases of Nedd4 family were co-transfected with Myc-SRSF5 into HEK293T cells, Expression levels of SRSF5 were analyzed by immunoblotting analysis. **c**, Knockdown of Smurf1 had no significant effect on SRSF5 mRNA expression level in A549 and H358 cells. Smurf1 knockdown efficiency was measured by qPCR (*$P$ < 0.05; n.s: no significance, Student's t-test). **d**, Immunoblotting analysis of SRSF5 and Smurf1 in seven representative tissues from adult *Smurf1*$^{+/+}$ and *Smurf1*$^{-/-}$ littermates. **e**, Analysis of SRSF5 mRNA levels in *Smurf1*$^{+/+}$, *Smurf1*$^{-/-}$ and *Smurf1*$^{+/-}$ MEFs. (NS: no significance, Student's t-test). **f**, E3 ligase activity is not a prerequisite condition for the interaction between Smurf1 and SRSF5. Flag-WT or C699A (CA) Smurf1 were co-expressed with Myc-SRSF5 in HEK293T cells. Smurf1 was immunoprecipitated, and SRSF5 protein was detected by immunoblotting analysis. **g**, **h**, Mapping of SRSF5 and Smurf1 binding regions. Coimmunoprecipitation assays were performed in the HEK293T cells transfected with the indicated constructs including the deletion mutants., The HECT domain of Smurf1 was involved in the interaction with SRSF5 **(g)** and the RS domain of SRSF5 was essential for the binding towards Smurf1 **(h)**. **i,** The schematic diagram of Smurf1 and SRSF5 binding regions. **j**, The relative mRNA level of Smurf1 in response to various glucose concentration were determined by qPCR. **k**. The relative mRNA level of Smurf1 during glucose deprivation at indicated time points were determined by qPCR. **l**, Smurf1 ubiquitylates SRSF5 *in vitro*. Purified HA–ubiquitin, E1, E2 (UbcH5c), bacteria-expressed and purified Smurf1 and SRSF5 were mixed for *in vitro* ubiquitylation assays and immunoblotted with anti-HA. **m**, The HECT domain of Smurf1 is both necessary and sufficient for the ubiquitylation of SRSF5 in cultured cells. HA-Ub, Flag-Smurf1 and Myc-SRSF5 were co-transfected into HEK293T cells, and treated with MG132. Ubiquitylated SRSF5 was immunoprecipitated with anti-HA antibody and analyzed by immunoblotting analysis. Data are representative of three independent biological replicates (**c, e, j, k**; mean and s.e.m., n = 3).

# Supplementary Figure 7. Acetylation of SRSF5 protects it from degradation

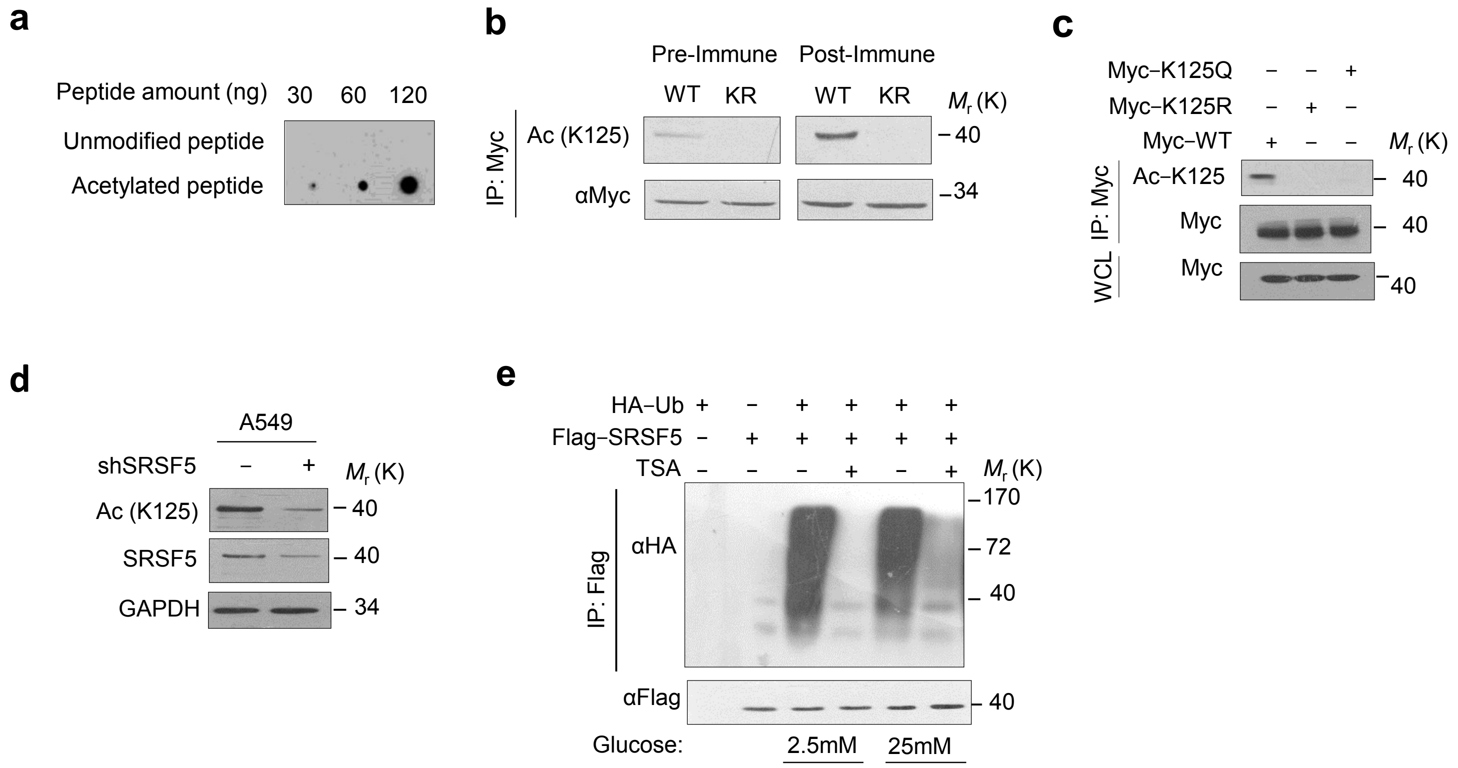


**Supplementary Figure 7 Acetylation of SRSF5 protects it from degradation**

**a**-**d**, Evaluation of the specificity for SRSF5-Ac K125 antibody. **a**, The specificity of the antibody against Ac-K125 was determined by dot blot. Nitrocellulose membrane was spotted with different amounts of either acetyl-K125 peptide or unmodified peptide and probed with anti-Ac-K125 antibody.

**b**-**c**, Myc-tagged SRSF5 WT or K125R were transfected into HEK293T cells and acetylation of each purified protein was measured by immunoblotting analysis using pre-immmune serum **(b)** or anti-Ac-K125 antibody **(c)**. **d**, A549 cells treated with or without SRSF5 shRNA were collected and subjected to immunoblot analysis, the endogenous Ac-K125 level were determined. **e**, TSA reduces SRSF5 ubiquitylation at various glucose concentrations. Flag-tagged SRSF5 was co-transfected with HA-tagged ubiquitin into HEK293T cells maintained under 2.5 mM or 25 mM glucose with or without TSA as indicated. Ubiquitylation of purified proteins was analyzed. Data are representative of three independent biological replicates.

# Supplementary Figure 8. HDAC1 deacetylates SRSF5 upon low glucose

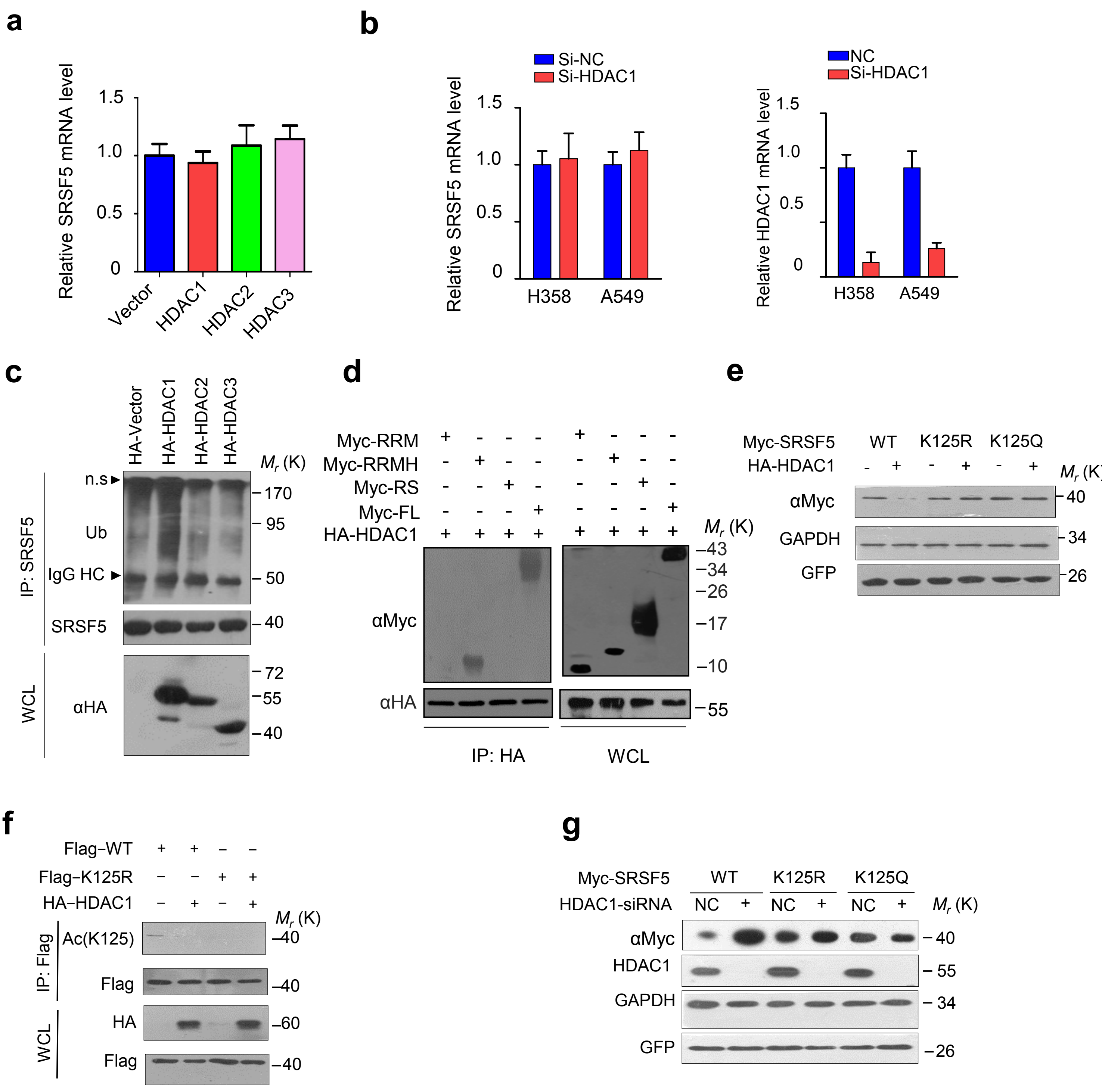


**Supplementary Figure 8 HDAC1 deacetylates SRSF5 upon low glucose**

**a**, Overexpression of HDAC1 had minor effect on SRSF5 mRNA expression level. Quantitative-PCR analysis of SRSF5 mRNA level was performed upon different groups of transfection. **b**, The mRNA level of SRSF5 remains unchanged upon HDAC1 knockdown. Quantitative PCR analysis of SRSF5 mRNA level were performed upon different treatment (**P* < 0.05, ns: no significance, Student's t-test). **c**, HDAC1 increased endogenous ubiquitylation of SRSF5. **d**, The RRMH domain of SRSF5 was the perquisite binding region of HDAC1. Coimmunoprecipitation assays were performed with the indicated SRSF5 truncates and HDAC1. **e**, Ectopic expression of HDAC1 decreased acetylation of wild-type, but not the K125R mutant of SRSF5. **f**, HDAC1 deacetylases SRSF5 at K125 in cells. Flag-tagged SRSF5 and K125R mutant were co-transfected with HA tagged HDAC1. Protein acetylation was determined by immunoblotting. **g**, HDAC1 knockdown increases the exogenous protein level of WT-type but not the K125R orK125Q mutant. Myc-Tagged WT, K125R, K125Q SRSF5 were co-transfected with HA-HDAC1 into HEK293T cells. The protein level of SRSF5 was determined by immunoblotting analysis. Data are representative of at least three independent biological replicates (**b**; mean and s.e.m., n = 3).

# Supplementary Figure 9. Uncropped scans of Western blots

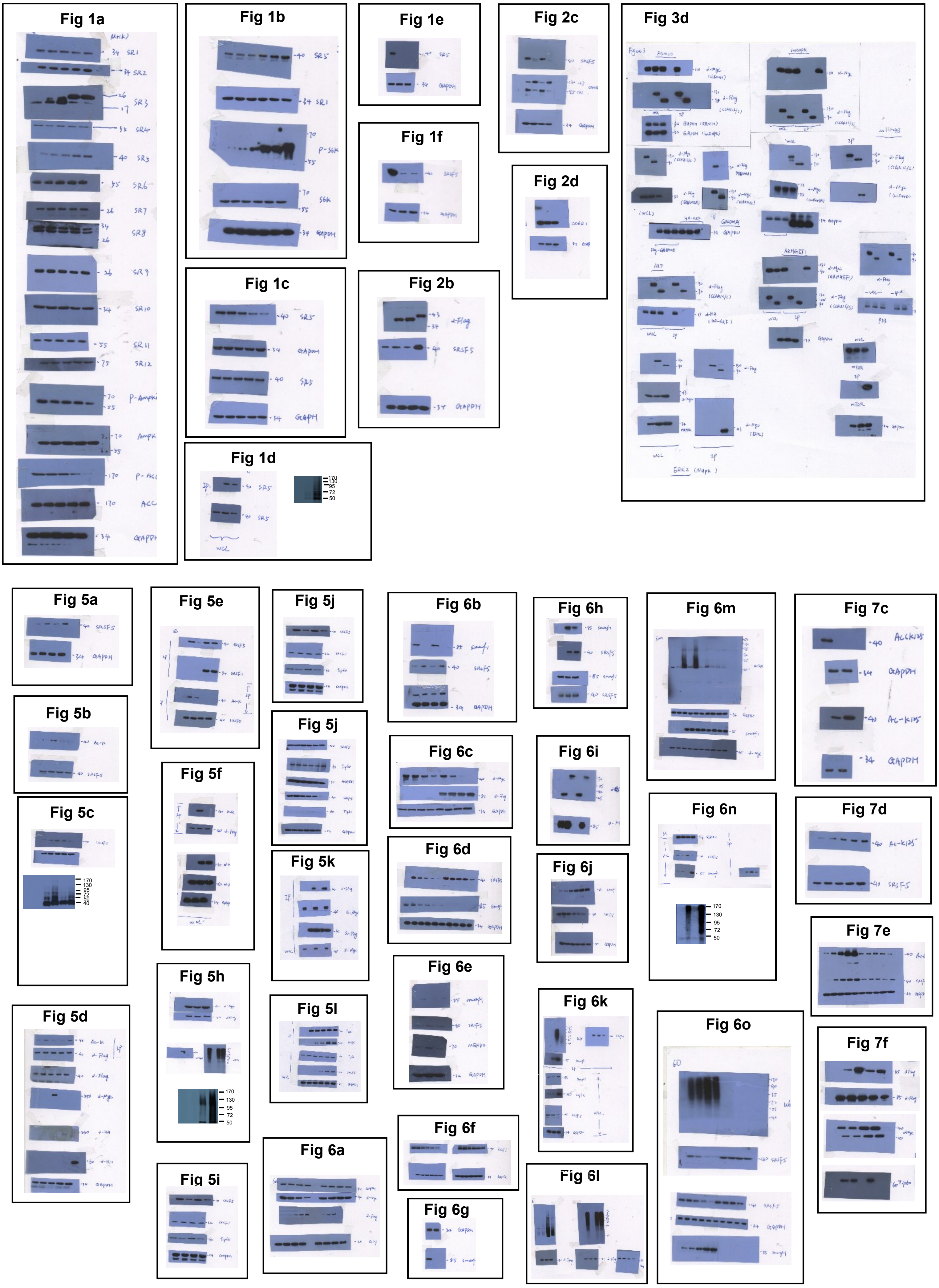

# Supplementary Figure 9. Uncropped scans of Western Blots (Continuation)

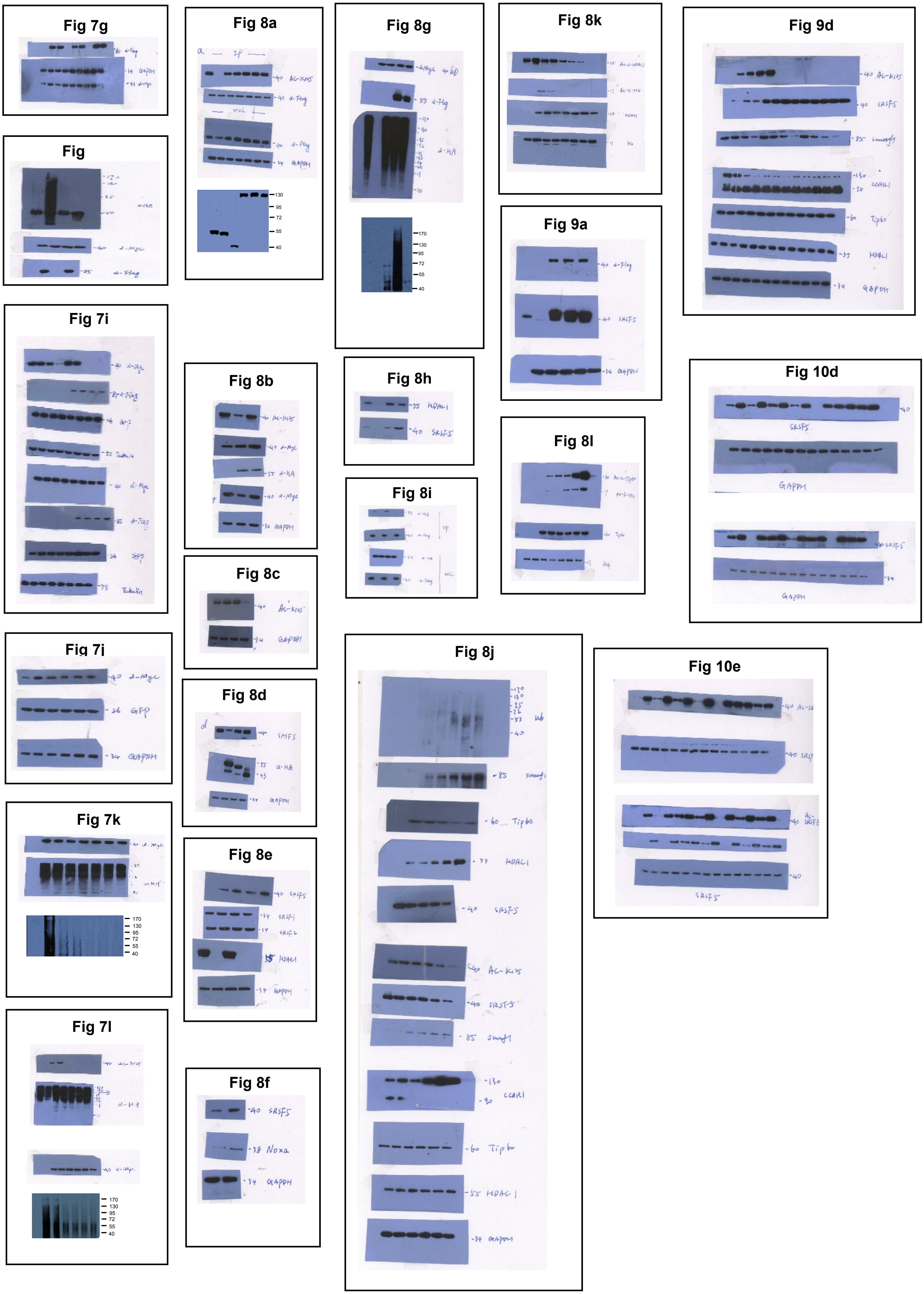

# Supplementary Figure 9. Uncropped scans of Western Blots (Continuation)

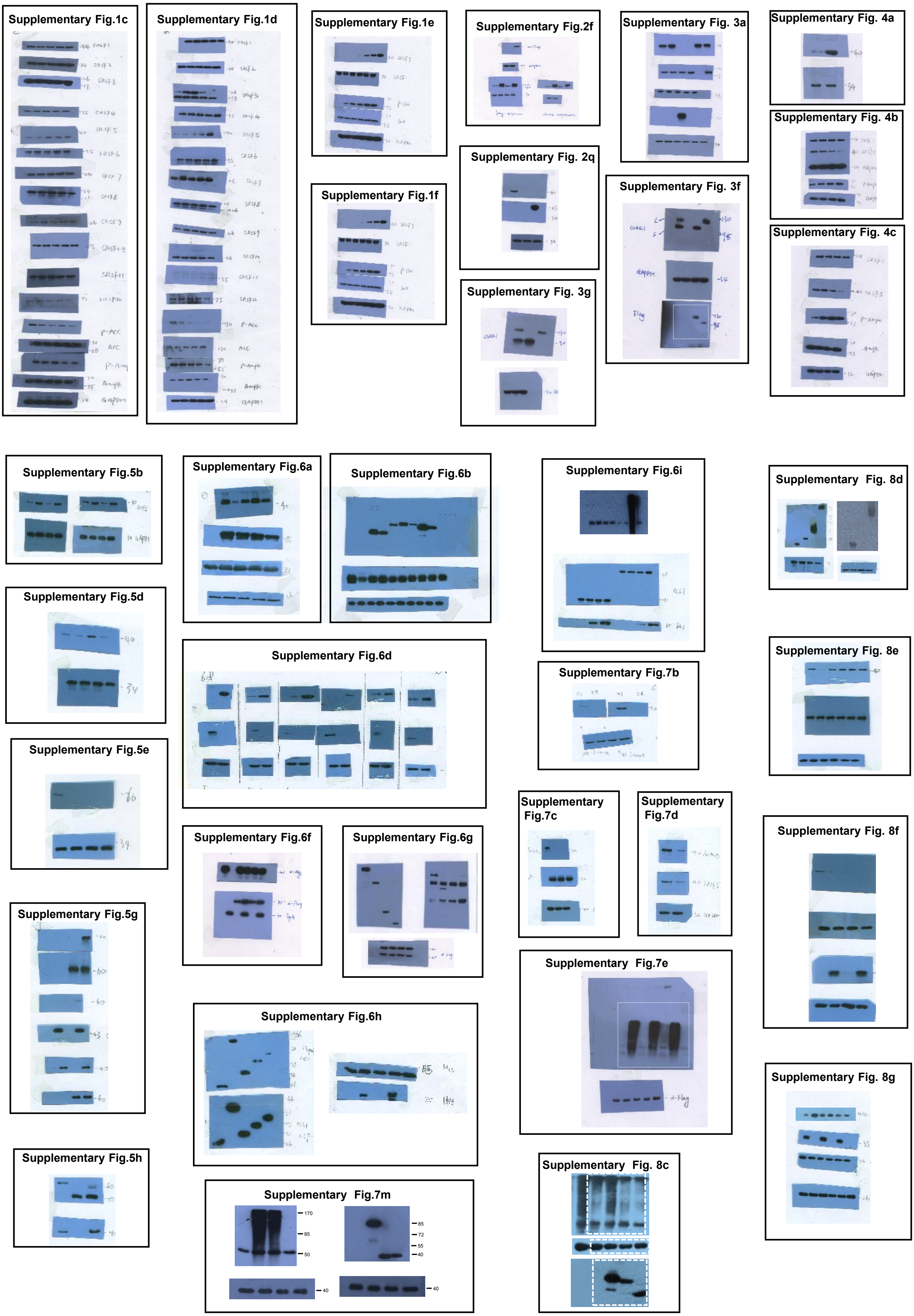

## Supplementary Table 1

Relationship between ratios of CCAR1 exons 15-22 exclusion/exons 15-22 inclusion in NSCLC samples and clinical pathological features of the individuals

| Clinical Characteristics | Non-increased CCAR1 Exclusion /inclusion exons15-22 (n=25) | Increased CCAR1 Exclusion/inclusion Exons15-22 (n=35) | Test of significance |
|---|---|---|---|
| Gender | | | |
| Male | 12 | 21 | c2 = 0.8485 |
| Female | 13 | 14 | *P** = 0.4339 |
| Age(years) | | | |
| ≤50 | 15 | 23 | *P** = 0.7869 |
| ≥50 | 10 | 12 | |
| TNM stage | | | |
| T1 | 3 | 5 | c2 = 0.07932 *P* = 0.9942 |
| T2 | 6 | 8 | |
| T3 | 7 | 10 | |
| T4 | 9 | 12 | |
| Tumor grade | | | |
| I | 12 | 5 | c2 = 10.97 *P* = 0.0119 |
| II | 8 | 10 | |
| III | 3 | 9 | |
| IV | 2 | 11 | |

Relationship between ratios of CCAR1 exons 15-22 exclusion/exons 15-22 inclusion in NSCLC samples and clinical pathological features of the individuals. Note: TNM, tumor/node/metastasis. Using SPSS V19.0 statistical software, a $\chi^2$-test (c2) or Fisher's exact test was performed to analyze the correlations of CCAR1 (exon15-exon22) mRNA ratios with clinical and pathological parameters. $P < 0.05$ was considered statistically significant. Tumor grade was based on TNM.
*Fisher's exact test.

## Supplementary Table 2

Relationship between ratios of increased SRSF5 / non-increased SRSF5 in NSCLC samples and clinical pathological features of the individuals

| Clinical Characteristics | Non-increasedSRSF5 (n=38) | Increased SRSF5 (n=22) | Test of significance |
|---|---|---|---|
| Gender | | | |
| Male | 13 | 10 | $c^2$ = 0.7452 |
| Female | 25 | 11 | *P** = 0.3880 |
| Age(years) | | | |
| ≤50 | 24 | 9 | *P** = 0.1133 |
| ≥50 | 14 | 13 | |
| TNM stage | | | |
| T1 | 2 | 1 | $c^2$ = 0.7749<br>*P* = 0.8554 |
| T2 | 7 | 5 | |
| T3 | 11 | 8 | |
| T4 | 18 | 8 | |
| Tumor grade | | | |
| I | 12 | 2 | *P** = 0.0327 |
| II | 10 | 3 | |
| III | 9 | 6 | |
| IV | 7 | 11 | |

Relationship between ratios of increased SRSF5 / non-increased SRSF5 in NSCLC samples and clinical pathological features of the individuals. Note: TNM, tumor/node/metastasis. Using SPSS V19.0 statistical software, a $\chi^2$-test (c2) or Fisher's exact test was performed to analyze the correlations of SRSF5 expression levels with clinical and pathological parameters. $P < 0.05$ was considered statistically significant. Tumor grade was based on TNM.
*Fisher's exact test.

# Supplementary Table 3. List of primers used for plasmids construction

| Genes | Forward Primer | Reverse Primer |
|---|---|---|
| Myc-SRSF5 | 5'-GGAATTCGGATGAGTGGCTGTCGGGTATTC-3' | 5'-CCGGTACCTTAATTGCCACTGTCAACTGAT-3' |
| Flag-SRSF5 | 5'-GGAATTCCATGAGTGGCTGTCGGGTATTC-3 | 5'-CGCTCGAGTAATTGCCACTGTCAACTGA-3' |
| Myc-RRM | 5'-CTGAATTCCATGAGTGGCTGTCGGGTAT-3' | 5'-CAGGTACCTCACCGAGCCCTAGCATGTT-3' |
| Myc-RRMH | 5'-GCGAATTCCATGCGTCTTATAGTTGAGA-3' | 5'-CGGGTACCTCAAATTAATTTTATTTTTCT-3' |
| Myc-RS | 5'-CTGAATTCCATGAGTAGGTCAAGAAGCAG-3' | 5'-CAGGTACCTCATTAATTGCCACTGTCAA-3' |
| GST-SRSF5 | 5'-GGAATTCGATGAGTGGCTGTCGGGTATTC-3' | 5'-CGGGTACCTAATTGCCACTGTCAACTGAT-3' |
| GST-RRM | 5'-CTGAATTCCCATGAGTGGCTGTCGGGTAT-3' | 5'-CATCTCGAGTCACCGAGCCCTAGCATGTT-3' |
| GST-RRMH | 5'-GCGAATTCCCATGCGTCTTATAGTTGAGA-3' | 5'-CGCCTCGAGTCAAATTAATTTTATTTTTCT-3' |
| GST-RS | 5'-CTGAATTCCCATGAGTAGGTCAAGAAGCAG-3' | 5'-CGTCTCGAGTCATTAATTGCCACTGTCAA-3' |
| Myc-Tip60 | 5'-GGAATTCGGATGGCGGAGGTGGTGAGT-3' | 5'-AGCAGCGGCCGCTCACCACTTCCCCCTC-3' |
| His-Tip60$^{212\text{-}513}$ | 5'-GGAATTCGGATGGCAGTGGCAGCCCAG-3' | 5'-CGCTCGAGTAACTGTCCTCTGACAGTGT-3' |
| Flag-Tip60$^{G380E}$ | 5'-ACCTTCGGCGATGTCAGTATGAGA-3' | 5'-TCTCATACTGACATCGCCGAAGGT-3' |
| HA-HDAC1$^{H178Y}$ | 5'-GGCAGTGGTAGCACTTATAGTTACA-3' | 5'-TGTAACTATAAGTGCTACCACTCC-3' |
| Myc-SRSF5$^{K125R}$ | 5'-CTGGCAGGATCTCAGAGATTCATGAGACA-3' | 5'-GACCGTCCTAGAGTCTCTAAAGTACTCTGT-3' |
| Myc-SRSF5$^{K125Q}$ | 5'-CTGCAGGATCTCCAAGATTTCATGAGACA-3' | 5'-GACCGTCCTAGAGGTTCTAAAGTACTCTGT-3' |
| Myc-RBM10 | 5'-CGAATTCGGATGGAGTATGAAAGACGT -3' | 5'-ATAGCGGCCGCTCACTGGGCCTCGTTG-3' |
| Myc-hnRNPK | 5'- CGAATTCGGATGGAAACTGAACAGCCA-3' | 5'- ATAGCGGCCGCTTAGAAAAACTTTCCAG -3' |
| pQCXIH-SRSF5$^{WT}$ | 5'-GCAACGGTAATGAGTGGCTGTCGGGTATTC-3' | 5'-CCGGGATCCTAATTGCCACTGTCAACTGAT-3' |
| pQCXIH-SRSF5 $^{K125R}$ | 5'-CTGGCAGGATCTCAGAGATTCATGAGACA-3' | 5'-GACCGTCCTAGAGTCTCTAAAGTACTCTGT-3' |
| pQCXIH-SRSF5 $^{K125Q}$ | 5'-CTGGCAGGATCTCCAAGATTTCATGAGACA-3' | 5'-GACCGTCCTAGAGGTTCTAAAGTACTCTGT-3' |
| pQCXIH-CCAR1S | 5'-CGCCAGCAGACATGATCATC-3' | 5'-GATGATCATGTCTGCTGGCG-3' |
| pQCXIH-CCAR1L | 5'-TGAAGGCGTCGGCGGCGCAGCTTTCAT-3' | 5'-ATGAAAGCTGCGCCGCCGACGCCTTCA-3' |

# Supplementary Table 4. List of primers for real-time PCR and genotyping

| Genes | Forward Primer | Reverse Primer |
|---|---|---|
| GAPDH | 5’-GGGAAGGTGAAGGTCGGAGT-3’ | 5’-TTGAGGTCAATGAAGGGGTCA-3’ |
| Smurf1 | 5’-CTACCAGCGTTTGGATCTAT-3’ | 5’-TGTCTCGGGTCTGTAAACT-3’ |
| SRSF5 | 5’-ATGAGACATTCAAAGAGAACTTACT-3’ | 5’-CCGAATTCCTGCTACACGTCTACCTCC-3’ |
| SRSF5 (mouse) | 5’-CGATTGAACATGCCCGGGCT-3’ | 5’-CTGCCAGCTGACTCTTGAGG-3’ |
| Tip60 | 5’-AGGGCACCATCCCTTCTTT-3’ | 5’-GTTAGGAT-CAGGCCACATT3’ |
| HDAC1 | 5’-ATCAGAGCCACACTAAGTAG-3’ | 5’-GTGGAAGTGAGAAGATAACG-3’ |
| CCAR1L | 5’-GTTTCAATCCCGCCAACGCA-3’ | 5’-CCGAACACGTCTCCTGCTC-3’ |
| CCAR1S | 5’-ACTCCTTGTAGCGACCTGG-3’ | 5’-CCACGCTATGATCAGAGCTACG-3’ |
| ISG15 | 5’-GAGGCAGCGAACTCATCTTT-3’ | 5’-AGCATCTTCACCGTCAGGTC-3 |
| STAT1 | 5'-GTGGAAAGACAGCCCTGCA-3’ | 5'-ACTGGACCCCTGTCTTCAA-3' |
| IFN-γ | 5′-TTCCTTGATGGTCTCCACAC-3′ | 5′-GAATTGGAAAGAGGAGAGTGA-3′ |
| ATF3 | 5′-ATGATGCTTCAACACCCAGGC-3’ | 5’-TTAGCTCTGCAATGTTCCTTC-3’ |
| PARP10 | 5’-AGGCGGCTGAGGAGTTTCT-3’ | 5’-GGCGCTCTGTCCCAAAGAC-3’ |
| REC8 | 5’-TCCGCGTCTATTCTCAACAATG-3’ | 5’-GGATCTGGAGCATCTTCTAGGG-3 |
| TP53AIP1 | 5’-CACCCATCTACAGTCC-3’ | 5’-ACCGTAGCTGCCCTGGTAG-3’ |
| TP53INP2 | 5’-GACCTCAGCGAAGGGGAATTG-3’ | 5’-CTGCTCTGGTTCTTGGACCG-3’ |
| FOXO1 | 5’-TCGTCATAATCTGTCCCTACACA-3’ | 5’-CGGCTTCGGCTCTTAGCAA-3’ |
| BAX | 5’-CCCGAGAGGTCTTTTTCCGAG-3’ | 5’-CCAGCCCATGATGGTTCTGAT-3’ |
| Smad4 | 5’-CTCATGTGATCTATGCCCGTC-3’ | 5’-AGGTGATACAACTCGTTCGTAGT-3’ |
| Gadd45 | 5’- TGCGAGAACGACATCAACAT-3’ | 5’-TGCGAGAACGACATCAACAT-3 |
| TGFBR3 | 5’-GTGTTCCCTCCAAAGTGCAAC-3’ | 5’-AGCTCGATGATGTGTACTTCCT-3’ |
| EGR1 | 5’-CCACGCCGAACACTGACATT-3’ | 5’-GAGGGGTTAGCGAAGGCTG-3’ |
| TNFSF9 | 5’-GGCTGGAGTCTACTATGTCTTCT-3’ | 5’-ACCTCGGTGAAGGGAGTCC-3’ |
| PCNA | 5’-ACACTAAGGGCCGAAGATAACG-3’ | 5’-ACAGCATCTCCAATATGGCTGA-3’ |
| Myo9A | 5’-AGGATGATGGAACGGGGTTTT-3’ | 5’-TGCTTAAAGCGATTTCGTAGGT-3’ |
| FMN2 | 5’-CAGAGCCTCGGTGTTTTCCAA-3’ | 5’-GCACACTCGGTATCCGACAG-3’ |
| ZBTB20 | 5’-GATGGAGGACGATTACGACTACT-3’ | 5’-GGTGCCTATGGAGGAGCTG-3’ |
| MAP2K6 | 5’-GAAGCATTTGAACAACCTCAGAC-3’ | 5’-CCTGGCTATTTACTGTGGCTC-3’ |
| Smurf1 WT allele | 5’-GGATCTAAGACTGTTTCCATGC-3’ | 5’-CACTTTGGGTCCAGGGTG-3’ |
| Smurf1 KO allele | 5’-GGATGTTGACTGGGAAG -3’ | 5’-CGACACTGTGAAAAATG-3’ |
