# Supplementary material for: Mutually exclusive acetylation and ubiquitylation of the splicing factor SRSF5 control tumor growth
Source: Nat Commun. 2018 Jun 25;9:2464. doi: 10.1038/s41467-018-04815-3 (PMC6018636; doi:10.1038/s41467-018-04815-3)
Supplement: Supplementary file 2 — Description of Additional Supplementary Files [file 41467_2018_4815_MOESM2_ESM.pdf]

Description of Additional Supplementary Files

File Name: Supplementary Data 1
Description: List of CCAR1 interacting proteins in distinct isoforms.

File Name: Supplementary Data 2
Description: List of Differentially Expressed Genes (DEGs) in Sh-con, Sh-CCAR1L and Sh-CCAR1S cells.
